# Gene expression profiling of recipient immune cells induced by 7 × 19 CAR-T cell dosing in a syngeneic mouse model

**DOI:** 10.1371/journal.pone.0352813

**Published:** 2026-07-17

**Authors:** Yo Muraki, Yuumi Okuzono, Rina Kurisu, Akiko Abiru, Yukimi Sakoda, Koji Tamada, Chihiro Take

**Affiliations:** 1 Oncology Drug Discovery Unit Japan, Research, Takeda Pharmaceutical Company Limited, Fujisawa, Kanagawa, Japan; 2 Noile-Immune Biotech Inc., Minato-ku, Tokyo, Japan; 3 Research Institute for Cell Design Medical Science, Yamaguchi University, Ube City, Yamaguchi, Japan; 4 Department of Immunology, Yamaguchi University Graduate School of Medicine, Ube City, Yamaguchi, Japan; Indian Institute of Chemical Technology, INDIA

## Abstract

Chimeric antigen receptor (CAR)-T cell therapy is effective for hematologic malignancies; however, the response of solid tumors is limited because of the immunosuppressive tumor microenvironment, antigen heterogeneity, and lack of persistence of transferred T cells. To overcome these challenges, CAR-T cells expressing interleukin-7 and CC chemokine ligand 19 (7 × 19 CAR-T) were generated to achieve potent antitumor efficacy through the recruitment and proliferation of CAR-T cells and endogenous immune cells. To elucidate the underlying mechanism of 7 × 19 CAR-T cells against solid tumors, we analyzed the cellular composition and gene expression profiles of host immune cells following CAR-T cell infusion in a murine solid tumor model. Antihuman CD20 7 × 19 CAR-T cells were prepared using Thy 1.1 congenic mice and administered to C57BL/6N mice bearing subcutaneous MC38 tumors expressing human CD20. The tumors were harvested 4 days postinfusion to capture early immune responses before overt tumor regression. CD90.1- recipient immune cells were subjected to flow cytometry analysis, and transcriptomic changes were determined using AmpliSeq and single-cell RNA seq. An increase in recipient CD8 + T cells and macrophages was observed in the tumor of mice treated with 7 × 19 CAR-T cells, but not with conventional CAR-T. The expression of chemokines and genes associated with the inflammatory pathway was upregulated only in recipient immune cells of the 7 × 19 CAR-T-treated mice. Single-cell RNA-seq analysis revealed upregulation of pro-inflammatory genes and chemokines in the dendritic cell and monocyte/macrophage populations. These results indicate that 7 × 19 CAR-T cells initiate the early recruitment and activation of host immune cells, which contributes to their superior antitumor activity compared with conventional CAR-T cells.

## Introduction

Chimeric antigen receptor (CAR)-T cell therapy is a groundbreaking anticancer treatment that has demonstrated remarkable efficacy in hematologic malignancies [1,2]. For example, CD19 CAR-T and B-cell maturation antigen CAR-T have been approved for B-cell acute lymphoblastic leukemia, large B-cell lymphoma, follicular lymphoma, mantle cell lymphoma, chronic lymphocytic leukemia, and multiple myeloma [3]. This highlights the potential of autologous CAR-T cells to revolutionize cancer treatment by harnessing the body’s immune system to target and eliminate cancer cells. However, compared with hematologic malignancies, the application of CAR-T cell therapy to solid tumors is limited, which is primarily due to solid tumor antigen heterogeneity and an immunosuppressive tumor microenvironment [4]. The latter, which includes the expression of immune checkpoint molecules, such as programmed cell death ligand 1 (PD-L1) or programmed cell death protein 1 (PD-1), as well as inhibitory immune cells, such as regulatory T cells (Tregs) and myeloid-derived suppressor cells, is considered a significant barrier to the effectiveness of CAR-T therapy in solid tumors.

To achieve effective results against solid tumors, the development of so-called PRIME CAR-T cells represents a promising strategy. These cells are engineered to express additional functional interleukin (IL)-7 and CC chemokine ligand (CCL) 19 genes in addition to the CAR gene, which endows the CAR-T cells with the ability to control a patient’s immune system in conjunction with the transferred CAR-T cells [5,6]. IL-7 is involved in the survival of T cells [7], whereas CCL19 is a chemoattractant for dendritic cells (DCs) [8]. CAR-T cells expressing IL-7 and CCL19 (7 × 19) exhibited potent antitumor efficacy in a syngeneic solid tumor model [5] compared with conventional CAR-T cells. This was partially attributed to improved CAR-T cell survival and enhanced infiltration of host immune cells, such as DCs and T cells, into tumor tissues, which resulted from functional IL-7 and CCL-19 expression; however, details of the host immune cell crosstalk within the tumor microenvironment remain unclear. To gain insight into the interaction between 7 × 19 CAR-T cells and host immune cells and the phenotypic characteristics of tumor-infiltrating immune cells, we isolated immune cells from tumor tissues after CAR-T cell administration and subjected them to transcriptome profiling and gene expression analysis.

In this study, CAR-T cells were generated from the splenocytes of B6 Thy1.1 mice, which express CD90.1 antigen instead of CD90.2, and were administered to CD90.1-negative tumor-bearing mice. Tumors were collected 4 days after CAR-T cell administration to examine the early interactions and effects on the host immune cells following CAR-T treatment. AmpliSeq analysis was conducted on CD90.1- CD45 + immune cells infiltrating the tumor to identify gene expression changes in the recipient immune cells that are associated with functional responses to CAR-T cells. In addition, single-cell RNA seq was performed on CD90.1-negative recipient immune cells to identify the biological pathways in specific immune cell subsets following 7 × 19 CAR-T cell treatment.

## Materials and methods

### CAR-T preparation

CAR constructs were prepared as previously described [5]. The anti-human CD20 scFv was fused to the transmembrane domain of mouse CD8α, cytoplasmic domains of mouse CD28, 4–1BB, and CD3ζ to yield a third-generation CAR, which was subsequently cloned into the retroviral vector MSGV1. To co-express murine IL-7 and CCL19 with CAR, the foot-and-mouth disease virus 2A peptide sequence was intercalated between the three genes. The retroviral transduction of mouse T cells with the CAR-expressing vectors was done as previously described. GP2–293 packaging cells (Clontech Laboratories, Mountain View, CA) were transfected with the CAR-expressing and pCL-Eco retrovirus packaging plasmids using Lipofectamine Reagent (Thermo Fisher Scientific, Waltham, MA). After 24 h, the supernatants containing CAR-expressing retrovirus were collected.

Mouse T cells were isolated from the splenocytes of B6 Thy1.1 mice (Charles River Laboratories, Wilmington, MA). Mice aged 5–12 weeks were anesthetized using isoflurane (1%–5%) and sacrificed. The spleens and lymph nodes were collected and mechanically dissociated using glass slides in RPMI 1640 medium (Thermo Fisher Scientific). Following red blood cell lysis with ACK T lysis buffer (Thermo Fisher Scientific), T cells were isolated from the lymphocytes using a T cell isolation kit (Miltenyi Biotech, Bergisch Gladbach, Germany). They were then seeded into 24-well plates that were pre-coated with anti-CD3 antibody (clone 145-2C11, Thermo Fisher Scientific) at a density of 3 × 10^6^ cells/well. After culturing for 2 days in RPMI1640 supplemented with 10% fetal bovine serum (Biosera, Cholet, France), penicillin–streptomycin (FUJIFILM Wako Pure Chemical, Osaka, Japan), 50 µM of 2-ME (Thermo Fisher Scientific), 25 mM of HEPES (Sigma-Aldrich, St. Louis, MO), 100 U/mL of IL-2 (PeproTech, Cranbury, NJ), and 1 µg/mL of anti-CD28 antibody (BioLegend, San Diego, CA), the cells were harvested and re-seeded into fresh 24-well plates at a density of 1 × 10^6^ cells/well. The cells were then transduced with culture supernatant containing CAR-expressing retrovirus. After 2 days of infection, the CAR-T cells were harvested, and CD20 CAR expression was assessed by flow cytometry (NovoCyte Flow Cytometer Systems, Agilent Technologies, Santa Clara, CA) following biotin-protein L and streptavidin-BV421 staining. CAR-T cells were resuspended in HBSS (Thermo Fisher Scientific) and administered to tumor-bearing mice.

### In Vivo Antitumor Efficacy Study of CAR-T Cells

The Institutional Animal Care and Use Committee of Shonan Health Innovation Park approved the protocol of the in vivo study (approval number: AU-00020667). Humane endpoints, such as body weight loss (20% initial weight loss), deterioration of general condition, and tumor volume > 2000 mm^3^, were defined in the protocol to alleviate suffering in mice during the study. C57BL/6N mice (CLEA Japan, Tokyo, Japan) were inoculated with MC38-human CD20 (hCD20) cells for the in vivo study. MC38-hCD20 cells were cultured in DMEM (Thermo Fisher Scientific) containing 10% of fetal bovine serum (Biosera), and penicillin–streptomycin (FUJIFILM Wako Pure Chemical). After expansion, the cells were detached with 0.05% trypsin-EDTA (Thermo Fisher Scientific), harvested, and resuspended in HBSS (Thermo Fisher Scientific). The mice were subcutaneously inoculated with MC38-hCD20 cells under anesthesia with 1%–5% isoflurane. After 7 days, tumor size was measured using calipers, and the mice were randomized into study groups. Next, 50 mg/kg of cyclophosphamide was administered intraperitoneally. After 3 days, CAR-T cells (1 × 10^6^ cells in 0.5 mL per mouse) were infused via the tail vein, and tumor volume was monitored for 18 days at intervals of 3 or 4 days after CAR-T injection. During the study period, the general condition and body weight of the mice were monitored, and deterioration of the general condition or body weight loss was not observed. The mice, whose tumor volume reached 2000 mm^3^, were sacrificed using 1%–5% isoflurane on the day of the tumor size measurement and were not used for further observation. Supplementary Table S1 in S1 File presents the individual data for tumor volume. In accordance with humane endpoint criteria and animal ethics considerations, two mice were sacrificed on day 14, and 4 mice were sacrificed on day 18. After the experiment, the mice were euthanized using 1%–5% isoflurane.

### Flow Cytometry Analysis of Immune Cells in the MC38-hCD20 Syngeneic Model

For the analysis of tumor-infiltrated lymphocytes by flow cytometry, tumor tissues were harvested from mice 4 days following CAR-T cell administration. The mice were anesthetized with isoflurane (1%–5%) and sacrificed. A single-cell suspension was prepared using the Dri Tumor & Tissue Dissociation Reagent (BD Biosciences, Franklin Lakes, NJ), following the manufacturer’s instructions. Briefly, the tumors were minced with dissection scissors in 0.5 mL of DMEM at the bottom of a 5 mL conical tube, transferred to tubes containing the dissociation reagent, and incubated for 30 min at 37°C. After enzymatic digestion, the tissues were passed through a 70 µm cell strainer and washed with phosphate-buffered saline (PBS) containing 1% bovine serum albumin and 2 mM EDTA. The cells were centrifuged, and the supernatant was removed. The cells were counted and seeded in a V-bottom 96-well plate at 1 × 10^6^ cells/well. After washing with PBS, dead cells were stained using the Zombie Aqua Fixable Viability Kit (BioLegend). Surface marker staining was achieved in the presence of Fc block (BD Biosciences). Supplementary Table S2 in S1 File lists the antibodies used for surface antigen detection. The flow cytometry data were analyzed as described in Supplementary S1 Fig. Donor-derived CD90.1 + CAR-T cells were excluded from the analysis, and the immunophenotypes of the recipient immune cells, including the proportion of T cells, B cells, DCs, monocyte/macrophages, and T cell surface markers, were analyzed.

### AmpliSeq Analysis of Recipient Immune Cells in the MC38-hCD20 Syngeneic Model

Single-cell suspensions of tumor-infiltrated lymphocytes were prepared as described in above. CD45 + cells were enriched using the CD45 EasySep positive selection kit (STEMCELL Technologies, Vancouver, Canada), followed by depletion of CD90.1 + cells. Total RNA was extracted from the remaining recipient immune cells, complementary DNA (cDNA) libraries were prepared using the AmpliSeq Mouse Gene Expression Kit (Thermo Fisher Scientific), and sequencing was done using the Ion S5 Sequencer (Thermo Fisher Scientific). Raw sequencing data were initially processed using the Ion Torrent Server (Thermo Fisher Scientific) to generate gene count data. Additional data analysis was done using R [9]. The detailed procedures are provided in Supplementary S1 Protocol. All AmpliSeq data were deposited in the Gene Expression Omnibus (GEO) repository (http://www.ncbi.nlm.nih.gov/geo) (accession number: GSE313034).

### Single-cell RNA-Seq Analysis of Recipient Immune Cells in the MC38-hCD20 Syngeneic Model

Single-cell RNA-seq libraries were prepared using CD45 + tumor-infiltrating immune cells. Single-cell suspensions were prepared as described in the previous section. CD45 + cells were isolated using the EasySep CD45 positive selection kit (STEMCELL Technologies). For CITE-seq analysis, the cells were labeled with TotalSeq-B0380 anti-rat CD90/mouse CD90.1 (Thy1.1) antibody (BioLegend). Single-cell libraries were prepared from tumor tissue using the Chromium Single Cell 3’ Library Kit v3.1 (10X Genomics) following the manufacturer’s instructions.

The libraries were sequenced on a DNBSEQ G-400 system at 100,000 reads per cell depth. The initial data was processed using the Cell Ranger pipeline (version 4, 10X Genomics). CD90.1 + donor-derived immune cells were identified based on anti-CD90.1 antibody binding and excluded from the downstream analysis. Further analysis was conducted using the Seurat package (version 5) in R [10], as detailed in Supplementary Protocol S2. All single-cell RNA-seq data were deposited in the Gene Expression Omnibus (GEO) repository (http://www.ncbi.nlm.nih.gov/geo, accession number: GSE314885).

## Results

### The antitumor effect of 7 × 19 CAR-T compared with conventional CAR-T in MC38-hCD20 syngeneic models

**Fig 1:**
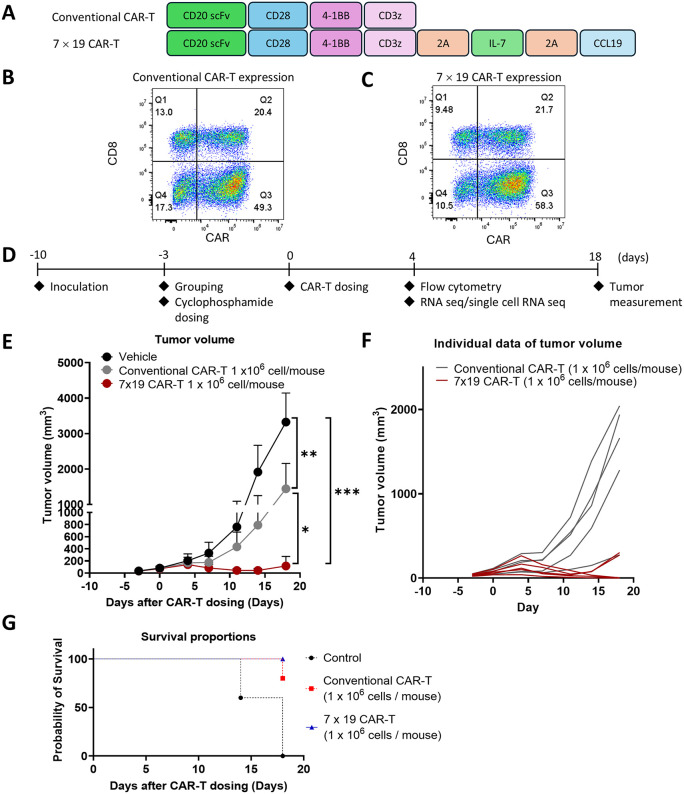
Overall study design and comparison of the antitumor effects of conventional CAR-T and 7 × 19 CAR-T in an MC38-hCD20-inoculated C57BL/6 syngeneic model. The antitumor effects of conventional CAR-T and 7 × 19 CAR-T were determined in MC38-hCD20-inoculated C57BL/6 mice. CAR-T cells were generated via transduction of mouse T cells with a CAR construct using a retroviral vector. (A) Constructs for conventional CAR-T and 7 × 19 CAR-T. (B-C) Flow cytometry analysis for confirmation of CAR expression in T cells following transduction. CD8 and CAR were stained for flow cytometry analysis. CAR expression was evaluated in the CAR+ cells among the total T cells. (B) CAR positivity of T cells transduced with conventional CAR-T. (C) CAR positivity of T cells transduced with 7 × 19 CAR-T. CAR expression was evaluated using flow cytometry following retrovirus vector transduction. (D) Study design. MC38-hCD20 cells were inoculated into C57BL/6N mice 10 days before CAR-T dosing. CAR-T was administered to the mice on Day 0. Tumor volume was monitored for 18 days following CAR-T injection. For transcriptome and flow cytometry analyses, the mice were sacrificed 4 days after CAR-T administration. (E) Tumor volume following CAR-T treatment. Average tumor volume of each group after administering vehicle, conventional CAR-T (1 × 10⁶ cells per mouse), or 7 × 19 CAR-T (1 × 10⁶ cells per mouse). Two mice in the control group, whose tumor volume reached 2000 mm^3^ on day 14, were sacrificed in accordance with the humane endpoint. Data are presented as the mean ± SD, n = 3–5. *: p ≤ 0.05, **: p ≤ 0.01, ***: p ≤ 0.001 by Tukey’s test. (F) Individual tumor volume data for mice treated with conventional CAR-T (1 × 10⁶ cells per mouse) and 7 × 19 CAR-T (1 × 10⁶ cells per mouse). (G) Survival of the mice was monitored from Days 0 to 18 after CAR-T dosing. Survival rate was calculated as the percentage of the mice that did not reach the humane endpoint. Mice that reached the humane endpoint (tumor volume > 2000 mm^3^) were sacrificed. The *p*-value from the log-rank test was < 0.05 (vehicle vs. conventional CAR-T, vehicle vs. 7 × 19 CAR-T).

To determine in vivo antitumor efficacy against human CD20-expressing subcutaneous MC38 tumors, we prepared third-generation murine anti-human CD20 CAR-T cells and 7 × 19 antihuman CD20 CAR-T cells using T cells derived from B6 Thy 1.1 mice. A schematic of the conventional and 7 × 19 CAR constructs is presented in Fig 1A. The 7 × 19 CAR construct was designed as a tricistronic cassette and encoded CAR, IL-7, and CCL19 separated by two 2A peptide sequences (Fig 1A). Flow cytometry indicated that CAR-T cell types exhibited over 70% CAR expression (Fig 1B, C).

MC38-hCD20 tumor-bearing syngeneic mice were intravenously injected with 1 × 10^6^ CAR+ cells of either conventional or 7 × 19 CAR-T cells (Fig 1D). After 7 days, the tumor volume was significantly reduced in both treatment groups (Fig 1E). At day 18, the antitumor effect was markedly increased in the 7 × 19 CAR-T cell group compared with the conventional CAR-T cell group. The average tumor volumes were 1440.7 ± 714.1 mm³ for the conventional CAR-T group and 117.2 ± 156.5 mm³ for the 7 × 19 CAR-T group (Fig 1E). Interestingly, complete tumor remission was observed in one mouse treated with 7 × 19 CAR-T cells. In contrast, no complete responses were observed in the conventional CAR-T group (Fig 1F). All mice in the vehicle group and one in the conventional CAR-T group reached the humane endpoint (tumor volume > 2000 mm^3^) during the study period (Fig 1G). All mice in the 7 × 19 CAR-T group did not reach the humane endpoint during the study period. Survival rate, defined as the percentage of mice that did not reach the humane endpoint, increased in the conventional CAR-T and 7 × 19 CAR-T groups compared with the control group (Fig 1G). The results indicate that 7 × 19 CAR-T cells have significantly greater antitumor efficacy compared with conventional CAR-T cells at the same dose (1 × 10^6^ cells), and this enhanced therapeutic effect of 7 × 19 CAR-T cells was evident from day 7 post-administration. To examine the early interactions between 7 × 19 CAR-T cells and host immune cells, before the onset of pronounced tumor regression, immunophenotypic and transcriptomic analyses were performed on tumor samples collected 4 days after CAR-T cell administration.

### Elevation of Monocyte/Macrophage, Recipient CCR7 + CD8 + T Cells and Central Memory T cell in the MC38-hCD20 Syngeneic Model Following 7 × 19 CAR-T Cell Treatment

To examine the early immune response to 7 × 19 CAR-T cells, we evaluated the immunophenotype of recipient immune cells within the tumor-infiltrating lymphocyte (TIL) population. CAR+ cells (1 × 10^6^ cells per mouse) representing conventional and 7 × 19 CAR-T cells derived from B6 Thy 1.1 mice, were administered intravenously into MC38-hCD20-inoculated C57BL/6 mice. After 4 days, tumors were harvested before the onset of measurable antitumor effects, and CD90.1- recipient immune cells were subjected to flow cytometry.

**Fig 2:**
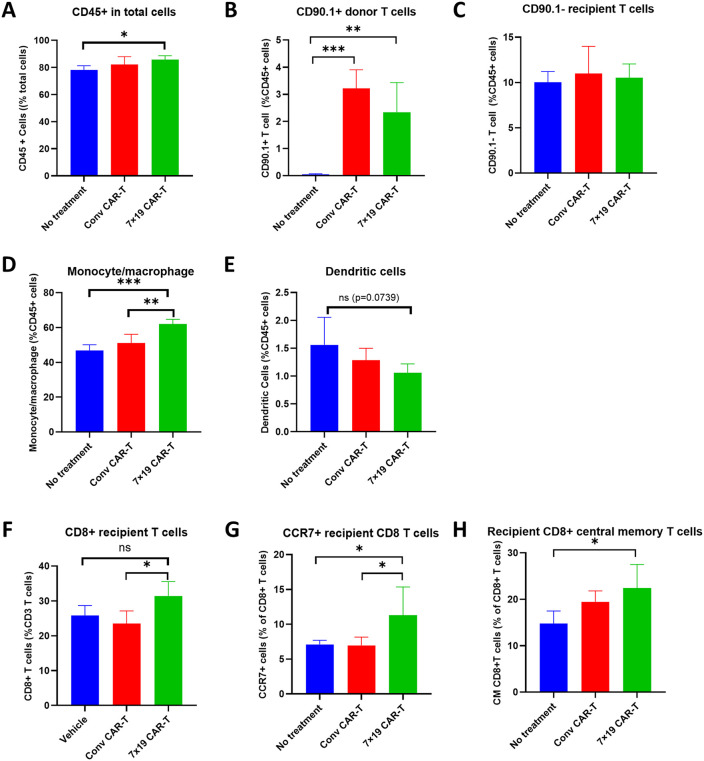
Flow cytometry analysis of recipient tumor-infiltrating lymphocytes in the MC38-hCD20 syngeneic model treated with conventional CAR-T and 7 × 19 CAR-T. Flow cytometry analysis was performed on tumors harvested 4 days after CAR-T dosing. CD45 + cells were gated from live cells. Then, CD90.1 CAR-T cells were gated as recipient CAR-T cells. CD45 + CD90.1 − cells were utilized to analyze recipient immune cells. B220 + cells were gated as B cells, CD11b+F4/80 + cells as monocyte/macrophage, CD11c+ cells as dendritic cells, and CD3 + CD8 + cells as CD8 T cells. CD44 + CD62L+ cells were gated to identify central memory CD8 T cells. (A) The percentage of recipient CD45 + cells among the total number of tumor cells. (B–E) The percentage of immune cell populations within CD45 + lymphocytes. Percentage of donor T cells (B), recipient T cells (C), recipient monocytes/macrophages (D), and recipient dendritic cells (E). (F–H) T cell subpopulations within recipient CD3 + T cells. Percentage of recipient CD8 + T cells (F), recipient CD8 + CCR7 + T cells (G), and recipient central memory T cells (H). Bars represent the mean ± SD, n = 5. *: p ≤ 0.05, **: p ≤ 0.01, ***: p ≤ 0.001 by Tukey’s test.

The number of harvested cells in tumor tissue was not significantly different among the study groups (the total number of harvested cells in the tumor tissues in the vehicle, conventional CAR-T, and 7 × 19 CAR-T groups were 224.5 ± 8.2, 250.7 ± 43.6, and 241.3 ± 24.9 × 10^4^ cells/tumor, respectively). The proportion of CD45 + lymphocytes was higher in the 7 × 19 CAR-T cell group compared with the vehicle control (Fig 2A). Donor-derived CD90.1 + CAR-T cells were detected within the TIL population, with comparable frequencies between the conventional and 7 × 19 CAR-T groups (3.2 ± 0.7% and 2.3 ± 1.1% of CD45 + cells, respectively) (Fig 2B). The percentage of recipient T cells remained consistent across all groups (Fig 2C); however, the proportion of monocytes/macrophage was significantly increased in the 7 × 19 CAR-T group compared with the vehicle and conventional CAR-T groups (Fig 2D). The proportion of DCs among CD45 + cells was comparable across all conditions (Fig 2E). Notably, the percentage of recipient CD8 + T cells among the total T cells was elevated in the 7 × 19 CAR-T group (Fig 2F), along with increased proportions of CCR7 + recipient T cells and central memory T cells (Fig 2G, H).

The results indicate that, despite a similar level of CAR-T cell infiltration at day 4 post-administration, 7 × 19 CAR-T treatment resulted in a distinct early remodeling of the tumor immune microenvironment. This included increased infiltration of monocytes/macrophages and CD8 + T cells, and enrichment of CCR7+ and central memory T cell subsets, which suggests the initiation of a 7 × 19 CAR-T-specific immunomodulatory effect before overt tumor progression.

### Upregulation of pro-inflammatory pathways in recipient immune cells by 7 × 19 CAR-T administration

To evaluate the transcriptome landscape of endogenous recipient immune cells following 7 × 19 CAR-T cell administration, AmpliSeq analysis was performed. CD45 + immune cells were isolated from tumor tissues, and CD90.1 + CAR-T cells were depleted to specifically analyze the recipient-derived immune cells.

**Fig 3:**
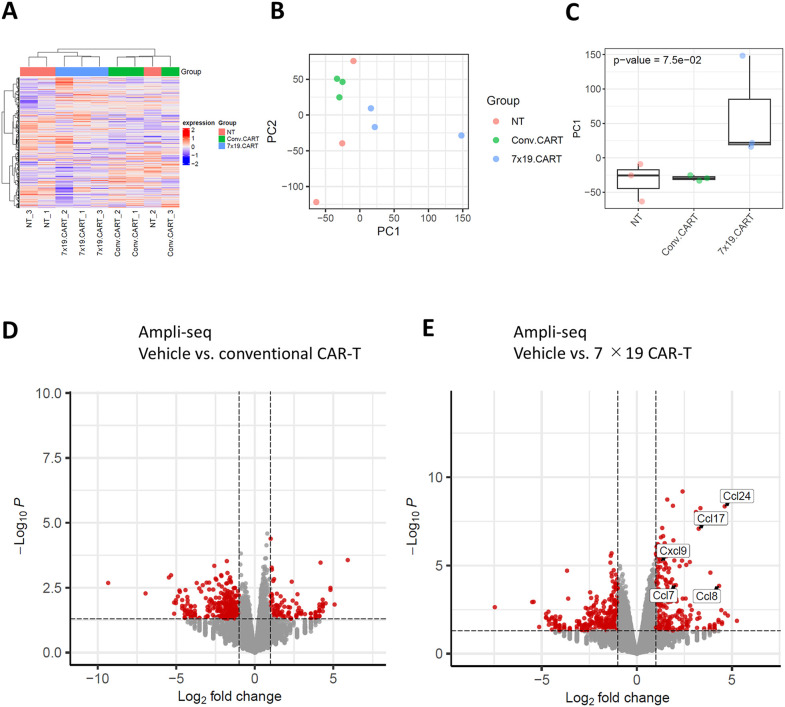
Principal component analysis of the AmpliSeq transcriptome data and identification of differentially expressed genes from recipient immune cells in the tumor following CAR-T treatment. AmpliSeq analysis was performed on CD90.1-CD45 + recipient immune cells following the depletion of CD90.1 donor cells. Visualization of gene expression data via heatmap and principal component analyses was conducted using the R software. (A) Heatmap showing individual gene expression among the samples. (B) Scatter plot of the first and second principal components in the principal component analysis. (C) Box plot of principal component 1 (PC1) distribution among the study groups. Differentially expressed genes (DEGs) between conventional CAR-T, 7 × 19 CAR-T, and vehicle groups were analyzed in CD90.1-CD45 + recipient immune cells. (D-E) Volcano plots showing gene expression changes following treatment with conventional CAR-T (D) and 7 × 19 CAR-T (E). Significant genes are defined by a p-value < 0.05 and an absolute fold-change >2.0. DEGs meeting these criteria are shown in red.

Transcriptomic profiles were visualized with a heatmap (Fig 3A). Principal component analysis (PCA) revealed that the gene expression profiles of the recipient immune cells in the 7 × 19 CAR-T group were distinct compared with those in the vehicle and conventional CAR-T groups (Fig 3B, C). Differentially expressed genes (DEGs) were identified and visualized using volcano plots (Fig 3D, E; Supplementary Tables S3 and S4 in S1 File). Multiple immune response genes were upregulated in the 7 × 19 CAR-T group. PCA analysis revealed that the PC1 values for the 7 × 19 CAR-T group differed significantly from those of the other groups. Chemokine-related genes, such as CCL24, CCL8, CCL17, CCL7, and CXCL9, were upregulated in response to the 7 × 19 CAR-T treatment (Fig 3D–E). The Ampli-seq data suggested that IL-7 and CCL19 expressions by CAR-T cells altered the transcriptional profile of the recipient tumor-infiltrating lymphocytes.

**Fig 4:**
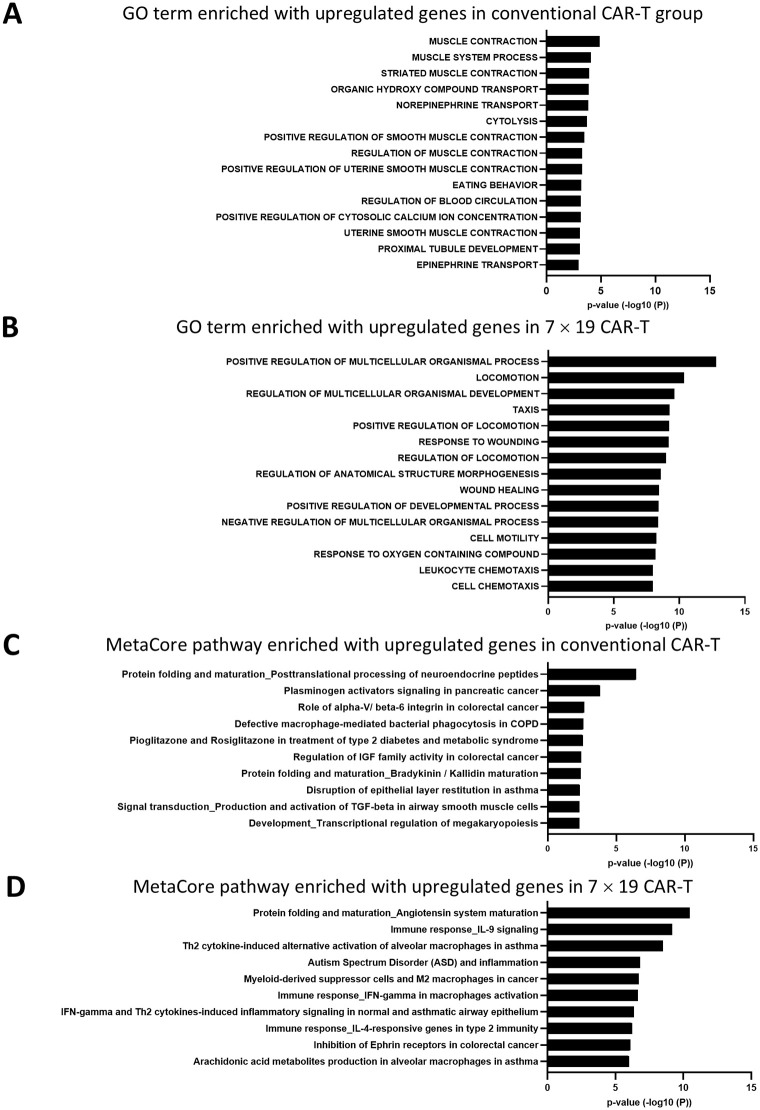
Pathway enrichment analysis of AmpliSeq transcriptome data from recipient immune cells in the tumors following CAR-T treatment. Pathway enrichment analysis was conducted using differentially expressed genes (DEGs) between the conventional CAR-T, 7 × 19 CAR-T, and vehicle groups using Gene Ontology (GO) term and MetaCore pathways. The top 15 enriched GO terms (A, B) and top 10 MetaCore pathways (C, D) were identified from upregulated genes in conventional CAR-T (A, C) or 7 × 19 CAR-T (B, D) groups. Bars represent the -log_10_ p-value for each pathway.

**Fig 5:**
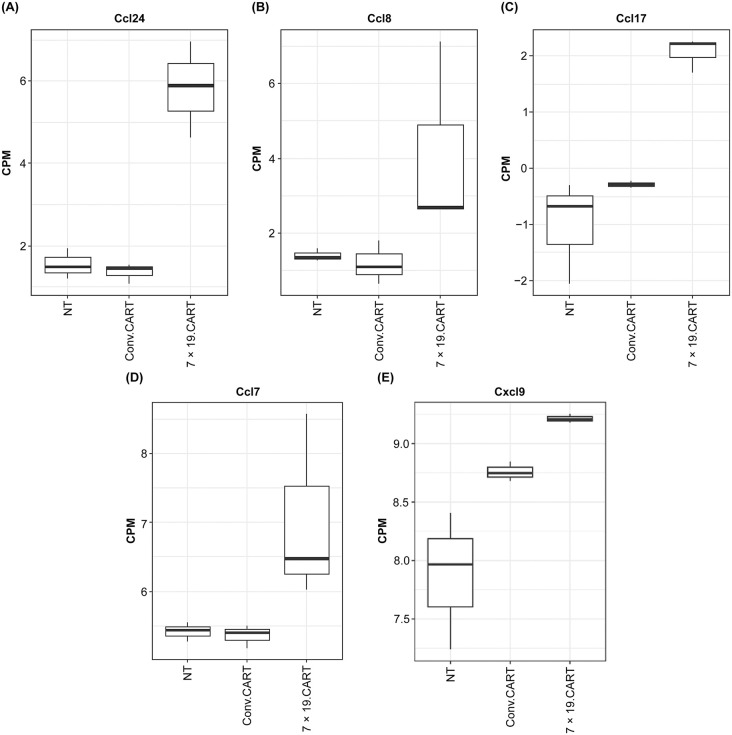
Chemokine gene expression analysis using AmpliSeq transcriptome data from recipient immune cells in tumors following CAR-T treatment. The expression levels of genes upregulated in the 7 × 19 CAR-T treatment were represented as a box plot. (A–E) Box plot analysis of chemokine genes induced by 7 × 19 CAR-T therapy. The X axis shows count per million (CPM). The expression CCL24 (A), CCL8 (B), CCL17 (C), CCL7 (D), and CXCL9 (E) is shown.

Pathway enrichment analysis was performed using Gene Ontology (GO) terms to further characterize the DEGs. GO terms associated with pro-inflammatory responses were significantly enriched in the 7 × 19 CAR-T group. The top 15 enriched pathways included “leukocyte chemotaxis” and “cell chemotaxis,” which were not observed in the conventional CAR-T group (Fig 4A, B). Further enrichment analysis using Metacore revealed that the “immune response IFN-gamma in macrophage activation” pathway was enriched in DEGs between the vehicle and the 7 × 19 CAR-T groups (Fig 4C, D). Moreover, chemokine-related genes, such as CCL24, CCL8, CCL17, CCL7, and CXCL9, were upregulated in response to 7 × 19 CAR-T treatment (Fig 5A–E). Overall, transcriptome analysis suggests that 7 × 19 CAR-T cells induce a robust pro-inflammatory response in recipient immune cells before the onset of measurable antitumor effects.

### Upregulation of pro-inflammatory pathway in the recipient DC, monocyte/macrophage, and T cells revealed by single-cell RNA-seq analysis

**Fig 6:**
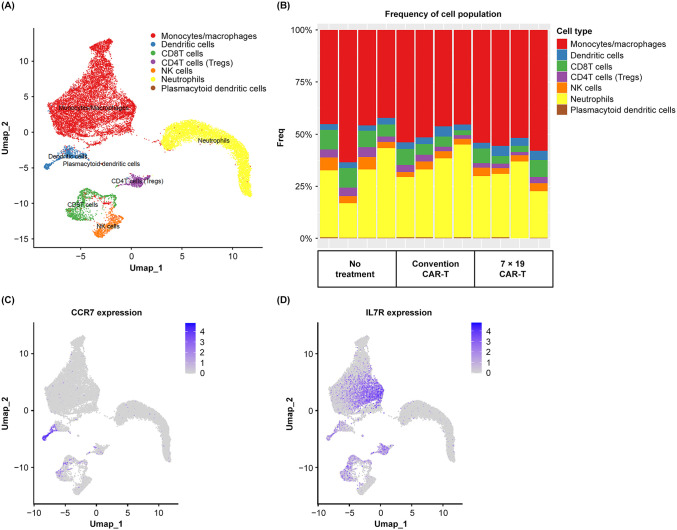
Single-cell RNA-seq analysis of recipient immune cells. Single-cell RNA-seq was performed on recipient immune cells. The single-cell RNA-seq data were analyzed using the R software. (A) Uniform Manifold Approximation and Projection (UMAP) plot of single-cell RNA-seq data from CD90.1-negative recipient immune cells. (B) The frequency of each cell type among the samples is shown as a percentage of the total cells. (C-D) Expression levels of receptors for CCL19 and IL-7. CCR7 and IL-7R expression levels are visualized in the UMAP plot.

Single-cell RNA-seq was conducted to evaluate the response of individual cell populations within recipient tumor-infiltrating lymphocytes. To specifically focus on recipient immune cells, donor-derived CD90.1 + T cells were excluded using Cellular Indexing of Transcriptomes and Epitopes by Sequencing (CITE-seq), with anti-CD90.1 antibodies conjugated to oligonucleotide barcodes. The recipient TILs were classified into seven distinct cell types, based on canonical marker gene expression, and visualized using Uniform Manifold Approximation and Projection plots (Fig 6A, B; Supplementary S2 and S3 Figs).

To determine the responsiveness of recipient cells to IL-7 and CCL19, the expression level of the IL-7 receptor (IL-7R) and CCR7 was assessed among the cell clusters (Fig 6C, D). IL-7R and CCR7 were prominently expressed in DCs and detected in CD8 + T cells. IL-7R expressions were also observed in monocytes/macrophages and CD4 + T cells.

**Fig 7:**
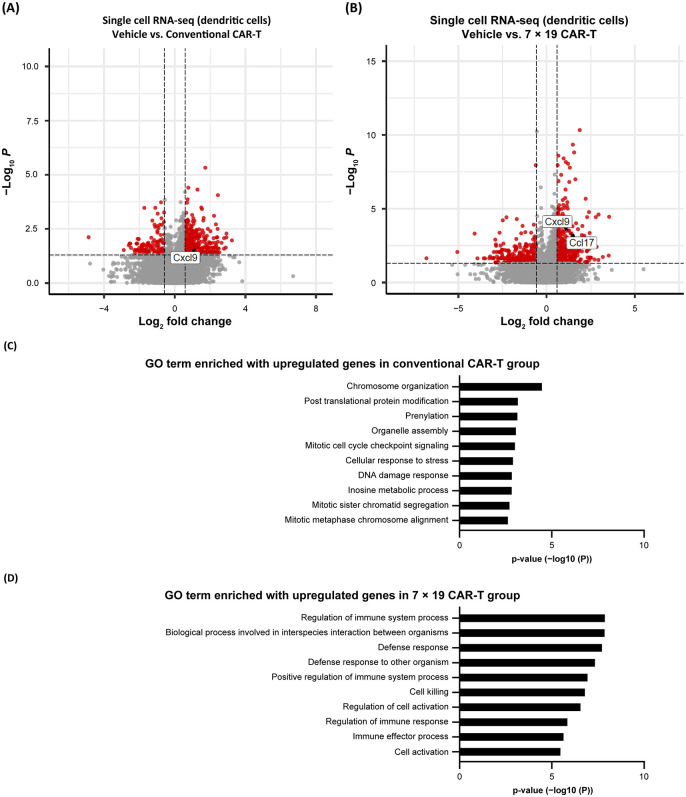
Differentially expressed genes (DEGs) in recipient dendritic cells. Single-cell transcriptomic analysis was conducted for recipient dendritic cells in the tumor tissues of mice treated with CAR-T. The DEGs in the control, conventional CAR-T, and 7 × 19 CAR-T groups were analyzed. (A, B) Volcano plots showing -log_10_ p-values versus log_2_ fold-changes from differential expression analysis between groups. DEGs were analyzed in recipient dendritic cells. Significant DEGs are defined by a p-value < 0.05 and an absolute fold-change > 1.5, with the most significant genes highlighted in red. (C-D) Top 10 Gene Ontology terms associated with the upregulated genes after CAR-T treatment. Enriched pathways from upregulated genes in the conventional CAR-T group (C) and the 7 × 19 CAR-T group (D) are shown. Bars represent the -log_10_ p-value for each pathway.

To examine the detail transcriptional response of specific cell types to 7 × 19 CAR-T cell treatment, DEGs were identified from single-cell RNA seq data. To capture broader transcriptomic changes, genes showing *p* < 0.05 were used, and 1.5-fold change was used as the criterion for DEGs in the single-cell RNA seq. In DCs, the DEGs between conventional and 7 × 19 CAR-T cell treatments were visualized using volcano plots (Fig 7A, B). Supplementary Tables S5 and S6 in S1 File list the genes. GO enrichment analysis revealed that pro-inflammatory pathways, such as “positive regulation of immune system process” and “immune effector process,” were enriched in the 7 × 19 CAR-T group, but not in the conventional CAR-T group (Fig 7C, D).

**Fig 8:**
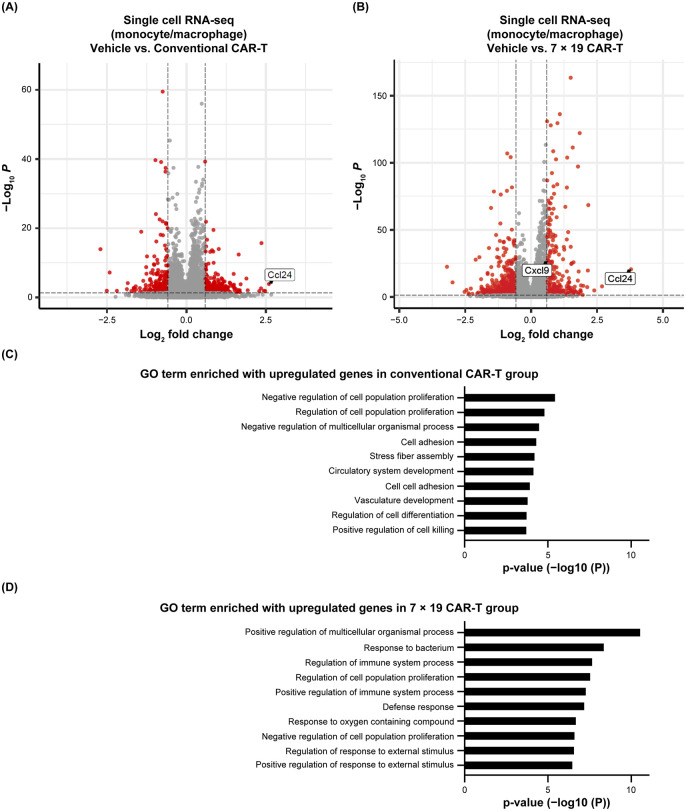
Differentially expressed genes (DEGs) in recipient monocytes/macrophages. Single-cell transcriptomic analysis was conducted for recipient monocytes/macrophages in the tumor tissues of mice treated with CAR-T. The DEGs in the control, conventional CAR-T, and 7 × 19 CAR-T groups were analyzed. (A, B) Volcano plots showing -log_10_ p-values versus the log_2_ fold-change from differential expression analysis between the groups. DEGs were analyzed in recipient monocytes/macrophages. Significant DEGs were defined by a p-value < 0.05 and an absolute fold-change > 1.5, with significant genes in red. (C-D) Pathway enrichment analysis based on the DEGs. Pathways enriched with upregulated DEGs in the conventional CAR-T group (C) and the 7 × 19 CAR-T group (D) are shown. Bars represent the -log_10_ p-value for each pathway.

Similarly, DEGs identified in the monocytes/macrophages were analyzed and visualized (Fig 8A, B; Supplementary Tables S7 and S8 in S1 File). GO analysis revealed enrichment of pathways associated with inflammation and cell proliferation, including “positive regulation of immune system process,” and “regulation of cell population proliferation,” in the 7 × 19 CAR-T group (Fig 8C, D).

**Fig 9:**
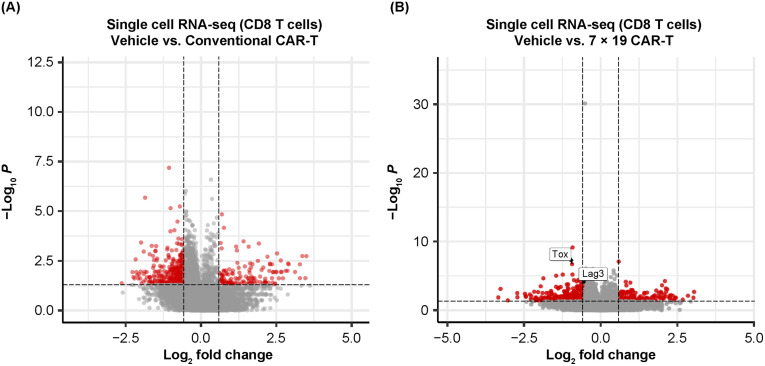
Differentially expressed genes (DEGs) in recipient CD8 + T cells. Single-cell transcriptomic analysis was conducted for recipient CD8 + T cells in the tumor tissues of mice treated with CAR-T. The DEGs in the control, conventional CAR-T, and 7 × 19 CAR-T groups were analyzed. (A, B) Volcano plots showing the log_10_ p-values versus log_2_ fold-changes based on differential expression analysis between the vehicle and conventional CAR-T groups (A), and the vehicle and 7 x 19 CAR-T groups (B). DEGs were analyzed in the recipient CD8 + T cells. Significant DEGs were defined by a p-value < 0.05 and an absolute fold-change > 1.5, with substantial genes highlighted in red.

The DEGs identified in CD8 + T cells were identified and summarized (Fig 9A, B; Supplementary Tables S9 and S10 in S1 File). Notably, the expression of exhaustion markers, such as TOX and the immune checkpoint molecule LAG3, was downregulated in CD8 + T cells from the 7 × 19 CAR-T treatment group compared with the conventional CAR-T group (Fig 9B, Supplementary Table S10 in S1 File).

Taken together, the results indicate that 7 × 19 CAR-T cell treatment induces a robust pro-inflammatory transcriptional program in recipient TILs. Genes associated with chemokine production and immune activation were differentially expressed in DCs, monocytes/macrophages, and CD8 + T cells, which highlights the immunomodulatory effects of 7 × 19 CAR-T cells before overt tumor regression.

## Discussion

This study examined the transcriptomic and immunophenotypic changes in recipient tumor-infiltrating lymphocytes (TILs) following short-term administration of murine anti-human CD20 7 × 19 CAR-T cells. As an initial response to 7 × 19 CAR-T treatment, significant gene expression changes were observed in the recipient immune cells, including monocytes/macrophages, DCs, and CD8 + T cells. These changes were characterized by activating pro-inflammatory pathways and upregulating chemokine genes, which likely contributed to the potent antitumor effect of the 7 × 19 CAR-T cells.

This is the first study to comprehensively examine the gene expression profiles and biological pathways upregulated by 7 × 19 CAR-T cell therapy. The transcriptomic landscape and enrichment pathways in recipient immune cells from 7 × 19 CAR-T-treated mice were distinct from those in the conventional CAR-T group. The results provide novel insight into the mechanisms underlying the enhanced antitumor effect of 7 × 19 CAR-T cells. Specifically, chemokines, including CCL24, CCL8, CCL17, CCL7, and CXCL9, were significantly induced, which provide valuable targets for future mechanistic studies. Of these, CCL24 expression was significantly increased in monocytes/macrophages. CCL24 is a ligand for CCR3 [11], which is expressed in eosinophils [11], which infiltrate solid tumors and contribute to antitumor immunity through interactions with various lymphocyte subsets, including T cells, NK cells, and innate lymphoid cells [12]. CCL24 induces macrophage chemotaxis [13]. Consistently, we observed an increased abundance of macrophages in tumors treated with 7 × 19 CAR-T. Clinical data indicate that high CCL24 expression in tumors correlates with a favorable prognosis in colorectal and gastric cancer [11], which suggests that CCL24 enhances antitumor immunity by modulating eosinophils and macrophage activity.

CCL17, a CCR4 ligand, was upregulated in DCs following 7 × 19 CAR-T treatment. CCR4 is expressed on TH2 cells, TH17 cells [14], and central memory T cells [15]. It has been implicated in antitumor immunity [16], and its overexpression in syngeneic tumor models enhances CD8 + T cells-mediated responses [17]. Bioinformatic analyses of clinical samples indicate that high CCL17 expression in cancer tissues positively correlates with pro-inflammatory gene signatures and a favorable prognosis, while negatively correlating with tumor progression signatures [18]. These results suggest that CCL17 upregulation in DCs plays a key role in enhancing the therapeutic efficacy of 7 × 19 CAR-T cells.

The other upregulated chemokines, CXCL9, CCL7, and CCL8, also support antitumor immunity. CXCL9, a ligand for CXCR3, promotes the migration of CD8 + T cells into inflamed tissues and tumors [19]. Its expression correlates positively with the abundance of intratumoral CD8 + cell abundance in humans [20]. CCL7 binds CCR1, CCR2, and CCR3, enhances CD8 + T cell expansion, and improves survival in preclinical tumor models [21]. Moreover, increased CCL7 expression in tumor tissue is associated with improved prognoses in patients with nonsmall cell lung cancer [21]. Similarly, CCL8 is a ligand for CCR8, which enhances immune cell infiltration and antitumor effects in syngeneic models [22]. These chemokines likely contribute to the robust antitumor effect observed with 7 × 19 CAR-T therapy.

In addition to chemokine modulation, we observed downregulation of exhaustion markers in recipient T cells. Specifically, the expression of LAG3, an immune checkpoint molecule [23], and thymocyte selection-associated HMG box (TOX), which is expressed on exhausted T cells [24], were decreased in CD8 + T cells from the 7 × 19 CAR-T group. This suggests that 7 × 19 CAR-T therapy suppresses early T cell exhaustion, thus enhancing long-term antitumor responses.

IL-7R was expressed in CD8^+^ T cells and monocytes/macrophages, whereas CCR7 was mainly expressed in dendritic cells. The potent antitumor effect of 7 × 19 CAR-T could be attributed to the close spatial configuration of IL-7 and CCL19 gradients, as CAR-T cells expressing either IL-7 or CCL19 alone did not demonstrate comparable antitumor efficacy [5]. Thus, the activation of pro-inflammatory pathways downstream of IL-7R and CCR7 within the tumor microenvironment may contribute to the enhanced antitumor activity of 7 × 19 CAR-T cells.

To elucidate early responses in the target cell populations, single-cell RNA-seq analysis was primarily conducted. In a previous study, the accumulation of recipient T cells and dendritic cells in tumor tissues was observed 21 days following the 7 × 19 CAR-T treatment [5]. Therefore, the early responses identified on Day 4 may represent initiating events that promote longer-term remodeling of the tumor microenvironment and ultimately contribute to the potent antitumor activity of 7 × 19 CAR-T cells. Future studies evaluating cytokines upregulated in plasma and tumor tissues, alongside histopathological analyses of tumor tissues at early time points, could provide further mechanistic insights into the antitumor effects of the 7 × 19 CAR-T treatment.

Our results provide insight into the early immune modulatory effects of 7 × 19 CAR-T cells on recipient immune cells. Transcriptomic analysis revealed the upregulation of chemokines and pro-inflammatory pathways that contribute to the superior antitumor efficacy of this therapy. These results also provide mechanistic insight into how 7 × 19 CAR-T cells may overcome immunosuppressive tumor microenvironments.

## Conclusion

This study elucidated the early immune responses triggered by 7 × 19 CAR-T therapy in recipient immune cells. This treatment activates the pro-inflammatory pathway, accompanied by the upregulation of chemokine genes associated with enhanced antitumor immunity. Our results suggest that the immunomodulatory effects of 7 × 19 CAR-T cells contribute to their remarkable therapeutic efficacy and offer a promising strategy for improving CAR-T activity in solid tumors.

## Supporting information

S1 Fig Gating strategy for flow cytometry analysis. Donor T cells were identified using CD90.1. Monocyte/macrophage cells were determined based on CD11b and F4/80 expression. Dendritic cells were identified based on CD11c expression. CD62L and CD44 expression were identified based on the phenotype of T cells.
(TIF)

S2 Fig Uniform Manifold Approximation and Projection (UMAP) of each treatment group. UMAP of the recipient tumor-infiltrated lymphocytes in the Vehicle (A), Conventional CAR-T (B), and 7 × 19 (C) groups.
(TIF)

S3 Fig Identification of the cell population in single-cell RNA seq using cell-specific markers. The expression of cell-type-specific markers was measured for each cluster.
(TIF)

S1 File Supplementary Tables. (Supplementary Table S1) Tumor volumes of individual mice in the control, conventional CAR-T, and 7 × 19 CAR-T groups. The tumor volume of each mouse at each time point. Mice No. 2 and 5 were sacrificed on day 14 because tumor volume reached to humane endpoint. Mice No. 1, 3, 4, and 10 were sacrificed on day 18, upon reaching the humane endpoint. (Supplementary Table S2) Antibodies used for flow cytometry analysis. Information for the antibodies used for flow cytometry analysis. (Supplementary Table S3) Differentially expressed genes (DEGs) in recipient immune cells after conventional CAR-T treatment. Upregulated or downregulated genes after conventional CAR-T dosing. The DEGs were defined by a p-value < 0.05 and an absolute fold-change > 2. (Supplementary Table S4) Differentially expressed genes (DEGs) in recipient immune cells after 7 × 19 CAR-T treatment. Upregulated or downregulated genes after 7 × 19 CAR-T dosing. The DEGs were defined by a p-value < 0.05 and an absolute fold-change > 2. (Supplementary Table S5) Differentially expressed genes (DEGs) in recipient dendritic cells after conventional CAR-T treatment. Upregulated or downregulated genes after conventional CAR-T dosing. The DEGs were defined by a p-value < 0.05 and an absolute fold-change > 1.5. (Supplementary Table S6) Differentially expressed genes (DEGs) in recipient dendritic cells after 7 × 19 CAR-T treatment. Upregulated or downregulated genes after 7 × 19 CAR-T dosing. The DEGs were defined by a p-value < 0.05 and an absolute fold-change > 1.5. (Supplementary Table S7) Differentially expressed genes (DEGs) in recipient monocyte/macrophage in the conventional CAR-T group. Upregulated or downregulated genes after 7 × 19 CAR-T dosing. The DEGs were defined by a p-value < 0.05 and an absolute fold-change > 1.5. (Supplementary Table S8) Differentially expressed genes (DEGs) in recipient monocyte/macrophage after 7 × 19 CAR-T dosing. Upregulated or downregulated genes after 7 × 19 CAR-T dosing. The DEGs were defined by a p-value < 0.05 and an absolute fold-change > 1.5. (Supplementary Table S9) Differentially expressed genes (DEGs) in recipient CD8 + T cells after conventional CAR-T dosing. Upregulated or downregulated genes after conventional CAR-T dosing. The DEGs were defined by a p-value < 0.05 and an absolute fold-change > 1.5. (Supplementary Table S10) Differentially expressed genes (DEGs) in recipient CD8 + T cells of the 7 × 19 CAR-T group. Upregulated or downregulated genes after 7 × 19 CAR-T dosing. The DEGs were defined by a p-value < 0.05 and an absolute fold-change > 1.5.
(XLSX)

S2 File Protocol. (DOCX)
