# Supplementary figures and images for: Gene expression profiling of recipient immune cells induced by 7 × 19 CAR-T cell dosing in a syngeneic mouse model
Source: PLoS One. 2026 Jul 17;21(7):e0352813. doi: 10.1371/journal.pone.0352813 (PMC13378967; doi:10.1371/journal.pone.0352813)

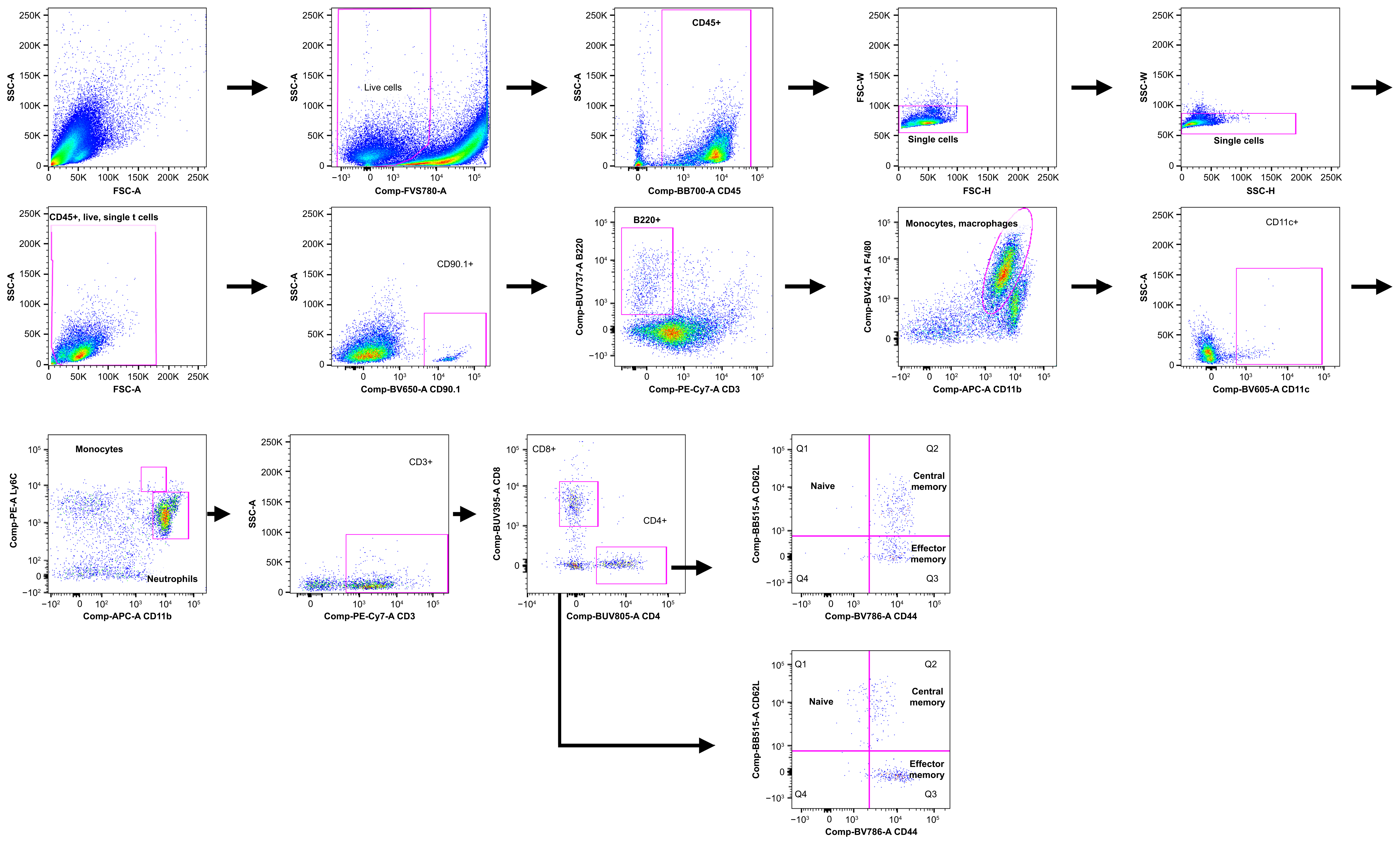

Supplement: S1 Fig — Donor T cells were identified using CD90.1. Monocyte/macrophage cells were determined based on CD11b and F4/80 expression. Dendritic cells were identified based on CD11c expression. CD62L and CD44 expression were identified based on the phenotype of T cells. (TIF) [file pone.0352813.s001.tif]

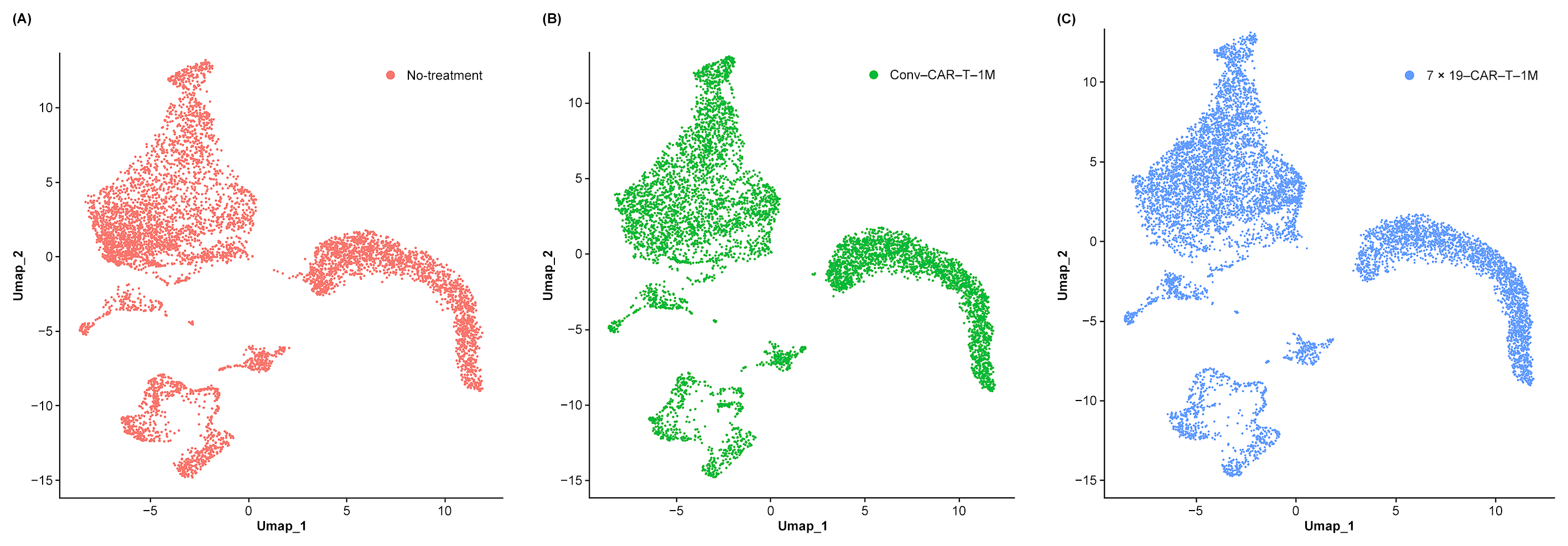

Supplement: S2 Fig — UMAP of the recipient tumor-infiltrated lymphocytes in the Vehicle (A), Conventional CAR-T (B), and 7 × 19 (C) groups. (TIF) [file pone.0352813.s002.tif]

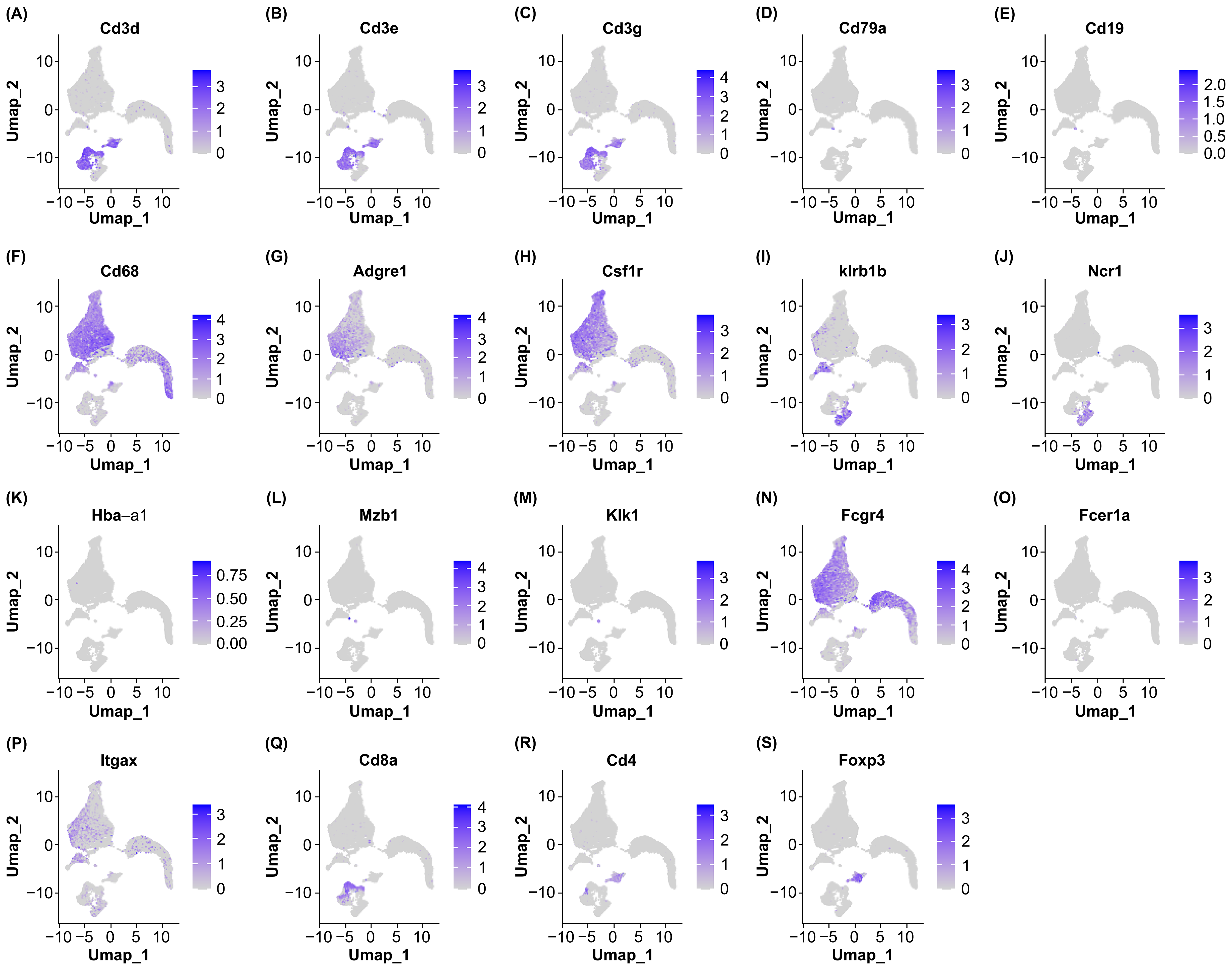

Supplement: S3 Fig — The expression of cell-type-specific markers was measured for each cluster. (TIF) [file pone.0352813.s003.tif]
