# Supplementary material for: Gene expression profiling of recipient immune cells induced by 7 × 19 CAR-T cell dosing in a syngeneic mouse model
Source: PLoS One. 2026 Jul 17;21(7):e0352813. doi: 10.1371/journal.pone.0352813 (PMC13378967; doi:10.1371/journal.pone.0352813)
Supplement: S2 File — (DOCX) [file pone.0352813.s005.docx]

# Supporting Information

# S1. Protocol Ampli-seq data analysis

Principal component analysis (PCA) was conducted using the 'prcomp' function of the stats package in R [1]. A test for equal means using a one-way layout was done to compare PC1 in each group using the "oneway.test" of the stats package in R. Visualization of the scatter plot and boxplot was done using the ggplot2 package in R. Heatmaps were generated using the ComplexHeatmap package in R [2]. The differentially expressed genes (DEGs) were identified based on the gene-wise negative binomial generalized linear model with the quasi-likelihood method using the edgeR package in R [3, 4]. Volcano plots for the DEG analysis were generated with the EnhancedVolcano package in R [5]. Pathway enrichment analyses of the DEGs were performed using the Correlation Engine software (Illumina, San Diego, CA, USA) using the 'Pathway Enrichment' workflow with the option "Select Biogroup to filter by Gene Ontology | GO" and Cortellis MetaCore software (Clarivate, Philadelphia, PA, USA) using 'Enrichment Analysis' workflow (default setting). Box plots were generated in R using the ggplot2 package [6] for visualization of gene expression levels. All the AmpliSeq data were deposited in the Gene Expression Omnibus (GEO) repository at <http://www.ncbi.nlm.nih.gov/geo> (GSE313034).

# S2 Protocol. Single-cell RNA seq data analysis

Data analysis was performed after deletion of low-quality cells (<200 genes/cell and >10% mitochondrial genes) and excluding doublet cells using the Seurat and DoubletFinder packages [7], respectively, in R. Count data were further normalized with the global-scaling normalization method, loge-transformation, and linear transformation to remove mitochondrial contamination, and the Hamming-corrected unique molecular identifier (UMI) count variations using the Seurat function "NormalizeData" and "ScaleData." The resulting top 2000 variable genes were used for PCA using the Seurat function "FindVariableFeatures" and "RunPCA." An elbow plot was generated by plotting the standard deviations of the principal components to identify the major principal components. These significant principal components were used for uniform manifold approximation and projection (UMAP) visualization, which was achieved through the Seurat functions "ElbowPlot" and "RunUMAP." The identification of cell types was done using these major principal components. First, cell similarities were calculated using a k-nearest neighbor graph based on the Euclidean distance in PCA space. The edge weights between any two cells were determined based on the shared overlap with their local neighborhoods using Jaccard's similarity, and facilitated by the Seurat function "FindNeighbors." Next, the cell populations were divided by clustering, which was achieved by assessing the similarities between cells using the Louvain algorithm and the Seurat function "FindClusters." Moreover, cell types within each cluster were identified by examining the cell type-specific marker expression profiles for each cluster using the Seurat function "FeaturePlot". Gene expression levels were visualized within each cell type using violin plots generated with the Seurat function "VlnPlot." To identify DEGs between groups within each cell type, the nonparametric Wilcoxon rank sum test was used. The Seurat function "FindMarkers" and Volcano plots for the DEG analysis were generated using “EnhancedVolcano” package [5] in R. Pathway enrichment analyses of the DEGs were performed using the Correlation Engine software using “Pathway Enrichment” workflow with the option "Select Biogroup filter by Gene Ontology | GO". Finally, bar and box plots were generated in R using the ggplot2 package [6] to visualize cell type frequencies and gene expression levels. All single-cell RNA-seq data were deposited in the GEO repository at <http://www.ncbi.nlm.nih.gov/geo> (GSE314885).

**References**

1. Ihaka R, Gentleman RR. A language for data analysis and graphics. J Comp Graph Stat. 1996;5: 299-314.

2. Gu Z, Eils R, Schlesner M. Complex heatmaps reveal patterns and correlations in multidimensional genomic data. Bioinformatics. 2016;32:2847-2849.

3. Robinson MD, McCarthy DJ, Smyth GK. edgeR: A Bioconductor package for differential expression analysis of digital gene expression data. Bioinformatics. 2010;26: 139-140.

4. McCarthy DJ, Chen Y, Smyth GK. Differential expression analysis of multifactor RNA-Seq experiments with respect to biological variation. Nucleic Acids Res. 2012;40: 4288-4297.

5. Blighe K, Rana S, Lewis M. EnhancedVolcano: Publication-ready volcano plots with enhanced colouring and labeling. 2018. Available from: <https://github.com/kevinblighe/EnhancedVolcano>.

6. Wickham H. Elegant graphics for data analysis. New York: Springer-Verlag.2016.

7. McGinnis CS, Murrow LM, Gartner ZJ. DoubletFinder: Doublet detection in single-cell RNA sequencing data using artificial nearest neighbors. cell syst. 2019;8: 329-337.
