# Supplementary material for: Gene expression profiling of recipient immune cells induced by 7 × 19 CAR-T cell dosing in a syngeneic mouse model
Source: PLoS One. 2026 Jul 17;21(7):e0352813. doi: 10.1371/journal.pone.0352813 (PMC13378967; doi:10.1371/journal.pone.0352813)
Supplement: Attachment — Submitted filename: PONE-D-26-00663 Comments_Khalid.docx [file pone.0352813.s006.docx]

The manuscript titled Gene Expression Profiling of Recipient Immune Cells Induced by 7 × 19 CAR-T Cell Dosing in a Syngeneic Mouse Model” by Yo Muraki et al. discusses the underlying mechanism of action of 7 x 19 CAR-T cells reported earlier. Earlier the 7 x 19 CAR-T cells had shown the dependency on resident immune cells for their activity. Therefore, the paper focusses on the cellular composition and gene expression patterns of the resident immune cells during early tumor induction phase in presence of 7 x 19 CAR-T cells.

The experimental approach is good but analysis of the data and presentation of figures may need further refinement. The manuscript needs the following revision for further improvement.

**Following minor changes need to be included,**

Page 3 Line 34: change transcriptomics to transcriptomic

Page 12 Line 313: I wonder if 1.5-fold change in expression level will have a significant biological impact. A more stringent criteria like at least 2-fold change is suggested to be used.

Page 19 Line 418-420: The transcriptomic landscape cannot be called markedly different with a threshold of 1.5 fold being set. Calling it a modest change would be more appropriate.

Page 19 Line 438: change “bioinformatics’ to “Bioinformatic”.

**Figures:**

In all figures a succinct experimental procedure should be mentioned for ease and clarity.

Figure 1 B and C: Label the diagram with Marker rather than the fluorophore.

Gating strategy should be succinctly mentioned in the figure legend

Figure 1 D: the schematic is not a true representation of study design as the tumor volume data points at 0 and 5 days are represented in Figure 1 E and F but the same is not indicated in the schematic Figure 1 D.

Figure 2: Gating strategy should be succinctly mentioned in the figure legend

E: please indicate the exact p value when comparing “no treatment” vs “7 x 19 CART-T”

F: please indicate whether statistics between “Vehicle” vs “7 x 19 CART-T” was significant.

Figure 3: It was difficult to make out anything out of the data presented given the low quality pictures provided.

Figure 4: It was difficult to make out anything out of the data presented given the low quality pictures provided.

Figure 5: abbreviation CPM on Y-axis should be expanded in the legend.

**Suggested Experiments:**

Overall, the paper gives an indication that treatment with 7 x 19 CAR-T cells induces a proinflammatory response in the tumors at the early stages with increased infiltration of immune cells. This fact can be cross-checked by using histopathological examinations of the tumor at early stages. Furthermore, validating the pro-inflammatory response through circulating cytokines in the mouse circulatory system would add a further degree of validation and confidence.
