# Supplementary material for: Gene expression profiling of recipient immune cells induced by 7 × 19 CAR-T cell dosing in a syngeneic mouse model
Source: PLoS One. 2026 Jul 17;21(7):e0352813. doi: 10.1371/journal.pone.0352813 (PMC13378967; doi:10.1371/journal.pone.0352813)
Supplement: Attachment — Submitted filename: Response to Reviewer.docx [file pone.0352813.s008.docx]

1/5/2026

Emily Chenette

Editor-in-Chief

*PLOS One*

Dear Editor:

Thank you for your comment on our submission entitled “Gene Expression Profiling of Recipient Immune Cells Induced by 7 × 19 CAR-T Cell Dosing in a Syngeneic Mouse Model.” We have revised the manuscript accordingly and present our response to the comment below.

**Response to reviewer**

Comment: A specific, scientific, and ethical justification for allowing larger tumor sizes than is typically considered acceptable.

Answer: The Institutional Animal Care and Use Committee (IACUC) of Shonan Health Innovation Park approved the protocol of the *in vivo* study (approval number: AU-00020667). The humane endpoint was defined as a tumor volume of 2000 mm^3^, assessed at 3- or 4-day intervals during tumor volume measurement. The study was conducted based on these criteria. Hence, the individual data for tumor volume were added in the Supplementary Table S1. The Supplementary Table numbers were revised due to the addition of a new supplementary table. After tumor volume measurement on day 14, the mice whose tumor volume reached 2000 mm^3^ (mice No. 2 and 5) were sacrificed. After tumor volume measurement on day 14, mice whose tumor volume reached 2000 mm^3^ (mice No. 1, 3, 4, and 10) were sacrificed. The animal study was performed according to the criteria for humane endpoint approved by IACUC. Revisions in the manuscript and Supplementary Tables were highlighted in yellow.

We look forward to your consideration and would be happy to provide any additional information if required.

Sincerely,

Chihiro Take

Oncology Drug Discovery Unit Japan, R&D, Research, Takeda Pharmaceutical Company Limited, 26-1, Muraoka-Higashi, 2-Chome, Fujisawa, 251-8555, Japan

Phone No: +81-466-32-1656

Fax No: +81-466-29-4412

Email Address: chihiro.akimoto@takeda.com
