# Supplementary material for: Gene expression profiling of recipient immune cells induced by 7 × 19 CAR-T cell dosing in a syngeneic mouse model
Source: PLoS One. 2026 Jul 17;21(7):e0352813. doi: 10.1371/journal.pone.0352813 (PMC13378967; doi:10.1371/journal.pone.0352813)
Supplement: Attachment — Submitted filename: Review 1.docx [file pone.0352813.s007.docx]

**Gene Expression Profiling of Recipient Immune Cells Induced by 7 × 19 CAR-T Cell Dosing in a Syngeneic Mouse Model**

1. In Figure 1, the authors show improved tumor reduction with 7×19 CAR-T cells compared to conventional CAR-T cells. However, no survival analysis is provided. Without survival data, it is difficult to assess the long-term therapeutic benefit of this treatment.
2. In Figure 2, the authors report increased percentages of CD8+ T cells and macrophages in the tumor microenvironment. However, only percentage data are presented, and absolute cell counts are not shown. This makes it unclear whether there is a true increase in immune cell numbers or only a relative change.
3. The study reports upregulation of multiple chemokines and immune-related pathways, but these findings are based only on transcriptomic analysis. No protein-level validation (such as ELISA) or functional assays are provided to confirm these observations.
4. The individual roles of IL-7 and CCL19 are not separately analyzed. Since both are expressed together in the CAR-T construct, it is unclear which factor is primarily responsible for the observed immunological effects.
5. The analysis is limited to an early time point (day 4 post-infusion). Long-term studies are required to evaluate persistence, durability of response, and overall therapeutic impact.
