# Supplementary material for: Gene expression profiling of recipient immune cells induced by 7 × 19 CAR-T cell dosing in a syngeneic mouse model
Source: PLoS One. 2026 Jul 17;21(7):e0352813. doi: 10.1371/journal.pone.0352813 (PMC13378967; doi:10.1371/journal.pone.0352813)
Supplement: Attachment — Submitted filename: Response to Reviewers_260522-2.docx [file pone.0352813.s009.docx]

5/22/2026

Emily Chenette

Editor-in-Chief

*PLOS One*

Dear Editor:

Thank you for your comment on our submission entitled “Gene Expression Profiling of Recipient Immune Cells Induced by 7 × 19 CAR-T Cell Dosing in a Syngeneic Mouse Model.” We have revised the manuscript accordingly and present our response to the comment below. The changes to the manuscript are highlighted in yellow in the Track Changes version of the manuscript file.

**Response to editor**

**Comment:**

1. Please ensure that your manuscript meets PLOS ONE's style requirements, including those for file naming. The PLOS ONE style templates can be found at

https://journals.plos.org/plosone/s/file?id=wjVg/PLOSOne_formatting_sample_main_body.pdf and

https://journals.plos.org/plosone/s/file?id=ba62/PLOSOne_formatting_sample_title_authors_affiliations.pdf

**Response:**

We confirmed that the manuscript meets PLOS ONE’s tyle requirements.

**Comment:**

2. To comply with PLOS One submissions requirements, in your Methods section, please provide additional information regarding the experiments involving animals and ensure you have included details on (1) methods of sacrifice, (2) methods of anesthesia and/or analgesia, and (3) efforts to alleviate suffering.

**Response:**

Thank you for this comment. To comply with the PLOS ONE requirements for animal experiments, we have revised the Methods section to include additional details on (1) the method of sacrifice/euthanasia, (2) anesthesia and/or analgesia, and (3) efforts to alleviate suffering. The mice were anesthetized with isoflurane (1%–5%) and euthanized under deep isoflurane anesthesia. We also added a description of our efforts to reduce pain and distress, including the use of predefined humane endpoint criteria approved by the Institutional Animal Care and Use Committee (IACUC); animals were euthanized immediately upon reaching these endpoints.

**Comment:**

3. Thank you for stating the following in the Competing Interests section:

“Yo Muraki, Yuumi Okuzono, Rina Kurisu, Akiko Abiru, and Chihiro Take are employees of Takeda Pharmaceutical Company Limited. Koji Tamada and Yukimi Sakoda hold stocks in Noile-Immune Biotech Inc. and receive remuneration from Noile-Immune Biotech Inc. In addition, Yukimi Sakoda received research funding from Noile-Immune Biotech Inc.”

We note that you received funding from a commercial source: Noile-Immune Biotech Inc

Please provide an amended Competing Interests Statement that explicitly states this commercial funder, along with any other relevant declarations relating to employment, consultancy, patents, products in development, marketed products, etc.

**Response:**

Thank you for this comment. We have amended the Competing Interests statement to explicitly declare the commercial funder, Noile-Immune Biotech Inc., and added this information to the Funding Statement. The revised Competing Interests statement now reads as follows:

YM, YO, RK, AA, and CT are employees of Takeda Pharmaceutical Company Limited. KT and YS hold stocks in Noile-Immune Biotech Inc. and receive remuneration from Noile-Immune Biotech Inc. In addition, Yukimi Sakoda received research funding from Noile-Immune Biotech Inc. This does not alter our adherence to PLOS ONE policies on sharing data and materials (as detailed online in our guide for authors <http://journals.plos.org/plosone/s/competing-interests>).

**Comment:**

Within this Competing Interests Statement, please confirm that this does not alter your adherence to all PLOS ONE policies on sharing data and materials by including the following statement: ""This does not alter our adherence to PLOS ONE policies on sharing data and materials.” (as detailed online in our guide for authors http://journals.plos.org/plosone/s/competing-interests). If there are restrictions on sharing of data and/or materials, please state these. Please note that we cannot proceed with consideration of your article until this information has been declared.

Please include your amended Competing Interests Statement within your cover letter. We will change the online submission form on your behalf.

**Response:**

Thank you for this request. We have added the following statement to the amended Competing Interests Statement in the revised cover letter: “This does not alter our adherence to PLOS ONE policies on sharing data and materials.”

**Comment:**

4. When completing the data availability statement of the submission form, you indicated that you will make your data available on acceptance. We strongly recommend all authors decide on a data sharing plan before acceptance, as the process can be lengthy and hold up publication timelines. Please note that, though access restrictions are acceptable now, your entire data will need to be made freely accessible if your manuscript is accepted for publication. This policy applies to all data except where public deposition would breach compliance with the protocol approved by your research ethics board. If you are unable to adhere to our open data policy, please kindly revise your statement to explain your reasoning and we will seek the editor's input on an exemption. Please be assured that, once you have provided your new statement, the assessment of your exemption will not hold up the peer review process.

**Response:**

Thank you for this comment. We have deposited the data in the NCBI Gene Expression Omnibus (GEO) under accession numbers GSE313034 and GSE314885. The datasets will be made publicly available upon acceptance of the manuscript for publication.

**Comment:**

5. Thank you for stating the following in the Competing Interests section:

“Yo Muraki, Yuumi Okuzono, Rina Kurisu, Akiko Abiru, and Chihiro Take are employees of Takeda Pharmaceutical Company Limited. Koji Tamada and Yukimi Sakoda hold stocks in Noile-Immune Biotech Inc. and receive remuneration from Noile-Immune Biotech Inc. In addition, Yukimi Sakoda received research funding from Noile-Immune Biotech Inc.”

We note that one or more of the authors are employed by a commercial company:

• Takeda Pharmaceutical Company Limited

• Noile-Immune Biotech Inc.

1) Please provide an amended Funding Statement declaring this commercial affiliation, as well as a statement regarding the Role of Funders in your study. If the funding organization did not play a role in the study design, data collection and analysis, decision to publish, or preparation of the manuscript and only provided financial support in the form of authors' salaries and/or research materials, please review your statements relating to the author contributions, and ensure you have specifically and accurately indicated the role(s) that these authors had in your study. You can update author roles in the Author Contributions section of the online submission form.

Please also include the following statement within your amended Funding Statement.

“The funder provided support in the form of salaries for authors [insert relevant initials], but did not have any additional role in the study design, data collection and analysis, decision to publish, or preparation of the manuscript. The specific roles of these authors are articulated in the ‘author contributions’ section.”

If your commercial affiliation did play a role in your study, please state and explain this role within your updated Funding Statement.

**Response:**

Thank you for this comment. We have amended the Funding Statement to declare the commercial affiliations and include a statement regarding the role of the funders. We added the following text to the revised cover letter:

The Takeda Pharmaceutical Company Limited provided support in the form of salaries for YM, YO, RK, AA, CT, Noile-Immune Biotech Inc provided support in the form of remuneration for KT, YS, but did not play any additional role in the study design, data collection and analysis, decision to publish, or preparation of the manuscript. The specific roles of these authors are articulated in the ‘Author Contributions’ section.

In addition, we have updated the author roles in the Author Contributions section of the online submission form to ensure they are specific and accurate.

**Comment:**

2) Please also provide an updated Competing Interests Statement declaring this commercial affiliation along with any other relevant declarations relating to employment, consultancy, patents, products in development, or marketed products, etc.

Within your Competing Interests Statement, please confirm that this commercial affiliation does not alter your adherence to all PLOS ONE policies on sharing data and materials by including the following statement: ""This does not alter our adherence to PLOS ONE policies on sharing data and materials.” (as detailed online in our guide for authors http://journals.plos.org/plosone/s/competing-interests). If this adherence statement is not accurate and there are restrictions on sharing of data and/or materials, please state these. Please note that we cannot proceed with consideration of your article until this information has been declared.

Please include both an updated Funding Statement and Competing Interests Statement in your cover letter. We will change the online submission form on your behalf.

**Response:**

Thank you for this request. We have included an updated Funding Statement and an updated Competing Interests Statement in our revised cover letter. The Competing Interests Statement also includes the following sentence: “This does not alter our adherence to PLOS ONE policies on sharing data and materials.”

**Comment:**

6. If the reviewer comments include a recommendation to cite specific previously published works, please review and evaluate these publications to determine whether they are relevant and should be cited. There is no requirement to cite these works unless the editor has indicated otherwise.

**Response:**

Thank you for this comment. The reviewer comments did not include any recommendation to cite specific previously published works. Therefore, no additional citations were added in response to this point.

**Comment:**

7. Please review your reference list to ensure that it is complete and correct. If you have cited papers that have been retracted, please include the rationale for doing so in the manuscript text, or remove these references and replace them with relevant current references. Any changes to the reference list should be mentioned in the rebuttal letter that accompanies your revised manuscript. If you need to cite a retracted article, indicate the article’s retracted status in the References list and also include a citation and full reference for the retraction notice.

**Response:**

Thank you for this comment. We carefully reviewed the reference list and confirmed that none of the cited papers have been retracted. Therefore, no changes were made to the reference list in response to this point.

**Response to reviewer**

Reviewer #1: The manuscript titled Gene Expression Profiling of Recipient Immune Cells Induced by 7 × 19 CAR-T Cell Dosing in a Syngeneic Mouse Model” by Yo Muraki et al. discusses the underlying mechanism of action of 7 x 19 CAR-T cells reported earlier. Earlier, the 7 x 19 CAR-T cells had shown the dependency on resident immune cells for their activity. Therefore, the paper focuses on the cellular composition and gene expression patterns of resident immune cells during the early tumor-induction phase in solid tumors, in the presence of 7 x 19 CAR-T cells to gain insights into the possible mechanism of action.

The experimental approach is good, but the data analysis and figure presentation may need further refinement. A more stringent criterion, like at least a 2-fold change, is suggested to be used for differentially expressed genes. The manuscript needs further refinement as suggested in the reviewer's report.

Following minor changes need to be included,

**Comment:**

Page 3 Line 34: change transcriptomics to transcriptomic

**Response:**

We changed “transcriptomics” to “transcriptomic.” (Page2 Line 35)

**Comment:**

Page 12 Line 313: I wonder if 1.5-fold change in expression level will have a significant biological impact. A more stringent criteria like at least 2-fold change is suggested to be used.

**Response:**

For the AmpliSeq analysis, we changed the threshold for differentially expressed genes from 1.5- to 2-fold. Accordingly, we updated the volcano plot and repeated the pathway enrichment analysis (Figs. 3 and 4). In the single-cell RNA seq study, we attempted to understand the whole cellular transcriptomic change following 7 × 19 CAR-T dosing and considered that the information including gene with low change is important for pathway enrichment analysis. Thus, we set 1.5-fold change as criteria in the single-cell RNA seq to capture broader transcriptomic changes. We described this point in the manuscript (Page 18, Line 401-405).

**Comment:**

Page 19 Line 418-420: The transcriptomic landscape cannot be called markedly different with a threshold of 1.5 fold being set. Calling it a modest change would be more appropriate.

**Response:**

We removed the word “markedly.” (Page 21. Line 474)

**Comment:**

Figures:

In all figures a succinct experimental procedure should be mentioned for ease and clarity.

**Response:**

We added a succinct description of the experimental procedure to all figure panels/legends for clarity.

**Comment:**

Figure 1 B and C: Label the diagram with Marker rather than the fluorophore. Gating strategy should be succinctly mentioned in the figure legend

**Response:**

We relabeled the diagram using marker names (instead of fluorophores) in Figure 1B and 1D and added a succinct description of the gating strategy to the figure legend.

**Comment:**

Figure 1 D: the schematic is not a true representation of study design as the tumor volume data points at 0 and 5 days are represented in Figure 1 E and F but the same is not indicated in the schematic Figure 1 D.

**Response:**

We revised the schematic and consolidated Figure 1E and 1F to accurately reflect the study design, including the tumor volume measurement time points.

**Comment:**

Figure 2: Gating strategy should be succinctly mentioned in the figure legend

E: please indicate the exact p value when comparing “no treatment” vs “7 x 19 CART-T”

F: please indicate whether statistics between “Vehicle” vs “7 x 19 CART-T” was significant.

**Response:**

We added a succinct description of the gating strategy to the Figure 2 legend.

In Figure 2E, we added the exact *p*-value for the comparison between “No treatment” and “7 × 19 CAR-T.”

In Figure 2F, we elucidated that the difference between “Vehicle” and “7 × 19 CAR-T” was not statistically significant and updated the figure accordingly.

**Comment:**

Figure 3: It was difficult to make out anything out of the data presented given the low quality pictures provided.

Figure 4: It was difficult to make out anything out of the data presented given the low quality pictures provided.

**Response:**

We revised Figures 3 and 4 to improve figure quality and readability.

**Comment:**

Figure 5: abbreviation CPM on Y-axis should be expanded in the legend.

**Response:**

We expanded “CPM” in the legend as “counts per minute.”

**Comment:**

Suggested Experiments:

Overall, the paper gives an indication that treatment with 7 x 19 CAR-T cells induces a proinflammatory response in the tumors at the early stages with increased infiltration of immune cells. This fact can be cross-checked by using histopathological examinations of the tumor at early stages. Furthermore, validating the pro-inflammatory response through circulating cytokines in the mouse circulatory system would add a further degree of validation and confidence.

**Response:**

Thank you for this comment regarding the study design. As you suggested, histopathological examination of tumors at early time points and measurement of circulating cytokines would provide additional validation and confidence in the proposed pro-inflammatory response. The present study focused on elucidating transcriptomic changes induced by the 7 × 19 CAR-T treatment; therefore, we did not perform these additional assays. We have added this limitation and the need for these follow-up analyses to the Discussion section (Page 23, Line 520-528).

Reviewer #2:

**Comment:**

1. In Figure 1, the authors show improved tumor reduction with 7 × 19 CAR-T cells compared to conventional CAR-T cells. However, no survival analysis is provided. Without survival data, it is difficult to assess the long-term therapeutic benefit of this treatment.

**Response:**

We added a survival analysis to Figure 1. As this study was designed to evaluate the early response to 7 × 19 CAR-T cells, it was ended 18 days following CAR-T dosing. The long-term therapeutic effects were reported in a previous study [reference]. In that study, 7 × 19 CAR-T cells demonstrated robust therapeutic efficacy, with survival in the syngeneic model sustained for up to 140 days [reference].

[reference] Adachi K, Kano Y, Nagai T, Okuyama N, Sakoda Y, Tamada K. IL-7 and CCL19 expression in CAR-T cells improves immune cell infiltration and CAR-T cell survival in the tumor. Nat Biotechnol. 2018;36(4):346–51. Epub 20180305. doi: 10.1038/nbt.4086. PubMed PMID: 29505028.

**Comment:**

2. In Figure 2, the authors report increased percentages of CD8+ T cells and macrophages in the tumor microenvironment. However, only percentage data are presented, and absolute cell counts are not shown. This makes it unclear whether there is a true increase in immune cell numbers or only a relative change.

**Response:**

In this study, we quantified the total cell numbers in the tumor tissue, and no significant differences were observed among the vehicle, conventional CAR-T, and 7 × 19 CAR-T groups (data added to the manuscript, Page 13, Lines 276–279). As the proportions of CD8+ T cells and macrophages increased despite similar total immune cell numbers across the groups, we interpreted these results as an increased abundance of CD8+ T cells and macrophages within the tumor immune compartment after the 7 × 19 CAR-T treatment.

**Comment:**

3. The study reports upregulation of multiple chemokines and immune-related pathways, but these findings are based only on transcriptomic analysis. No protein-level validation (such as ELISA) or functional assays are provided to confirm these observations.

**Response:**

This study focused on the characterization of transcriptomic changes after the 7 × 19 CAR-T treatment; therefore, we did not perform protein-level validation. We agree that follow-up studies measuring pro-inflammatory molecules at the protein level (e.g., by ELISA) would be valuable for further elucidating the mechanisms underlying the potent antitumor effects of 7 × 19 CAR-T cells.

**Comment:**

4. The individual roles of IL-7 and CCL19 are not separately analyzed. Since both are expressed together in the CAR-T construct, it is unclear which factor is primarily responsible for the observed immunological effects.

**Response:**

The study demonstrated a potent enhancement of the anti-tumor effect with IL-7 and CCL19 co-expression, whereas the expression of IL-7 and CCL19 alone did not produce a marked enhancement in anti-tumor efficacy ([reference], Supplementary Fig. 12). We conducted this study as the upregulated pathways under IL-7 and CCL19 co-expression are important for achieving potent anti-tumor effect. We considered that the entire immunological change could be important in the potent anti-tumor effect of 7 × 19 CAR-T.

[reference] Adachi K, Kano Y, Nagai T, Okuyama N, Sakoda Y, Tamada K. IL-7 and CCL19 expression in CAR-T cells improves immune cell infiltration and CAR-T cell survival in the tumor. Nat Biotechnol. 2018;36(4):346–51. Epub 20180305. doi: 10.1038/nbt.4086. PubMed PMID: 29505028.

**Comment:**

5 The analysis is limited to an early time point (day 4 post-infusion). Long-term studies are required to evaluate persistence, durability of response, and overall therapeutic impact.

**Response:**

To elucidate the early response to 7 × 19 CAR-T cells, we conducted transcriptomic analysis on day 4 post-infusion. In a previous study, long-term effects were reported, including the accumulation of dendritic cells and recipient T cells in tumor tissues, as well as prolonged survival [reference]. The increased chemokine production and upregulation of pro-inflammatory pathways observed in the present study may be associated with the previously reported long-term effects. Future studies evaluating the durability of these early responses (e.g., persistence of immune-cell infiltration and inflammatory signaling at later time points) will further elucidate the mechanisms underlying the potent antitumor efficacy of 7 × 19 CAR-T cells.

[reference] Adachi K, Kano Y, Nagai T, Okuyama N, Sakoda Y, Tamada K. IL-7 and CCL19 expression in CAR-T cells improves immune cell infiltration and CAR-T cell survival in the tumor. Nat Biotechnol. 2018;36(4):346–51. Epub 20180305. doi: 10.1038/nbt.4086. PubMed PMID: 29505028.

Sincerely,

Chihiro Take

Oncology Drug Discovery Unit Japan, R&D, Research, Takeda Pharmaceutical Company Limited, 26-1, Muraoka-Higashi, 2-Chome, Fujisawa, 251-8555, Japan

Phone No: +81-466-32-1656

Fax No: +81-466-29-4412

Email Address: chihiro.akimoto@takeda.com
