# Supplementary material for: Gene expression profiling of recipient immune cells induced by 7 × 19 CAR-T cell dosing in a syngeneic mouse model
Source: PLoS One. 2026 Jul 17;21(7):e0352813. doi: 10.1371/journal.pone.0352813 (PMC13378967; doi:10.1371/journal.pone.0352813)
Supplement: Peer review history

# Author comment: Author response to Decision Letter 0

**DOI:** 10.1371/journal.pone.0352813.r001

8 Jan 2026

Attachment Submitted filename: Response to Reviewer.docx

# Peer review: Decision Letter 0

**DOI:** 10.1371/journal.pone.0352813.r002

**Authors:** Srinivasa Reddy Bonam

9 Apr 2026

PONE-D-26-00663

Gene Expression Profiling of Recipient Immune Cells Induced by 7 × 19 CAR-T Cell Dosing in a Syngeneic Mouse Model

PLOS One

Dear Dr. Take,

Thank you for submitting your manuscript to PLOS ONE.After careful consideration, we feel that it has merit but does not fully meet PLOS ONE’s publication criteria as it currently stands. Therefore, we invite you to submit a revised version of the manuscript that addresses the points raised during the review process.

We have now secured two reviewers to comment on your study. The reviewers have provided their comments, which are attached for your consideration.

Please submit your revised manuscript by May 24 2026 11:59PM. If you will need more time than this to complete your revisions, please reply to this message or contact the journal office at plosone@plos.org. When you're ready to submit your revision, log on to https://www.editorialmanager.com/pone/ and select the 'Submissions Needing Revision' folder to locate your manuscript file.

Please include the following items when submitting your revised manuscript:

- A letter that responds to each point raised by the academic editor and reviewer(s). You should upload this letter as a separate file labeled 'Response to Reviewers'.
- A marked-up copy of your manuscript that highlights changes made to the original version. You should upload this as a separate file labeled 'Revised Manuscript with Track Changes'.
- An unmarked version of your revised paper without tracked changes. You should upload this as a separate file labeled 'Manuscript'.

If you would like to make changes to your financial disclosure, please include your updated statement in your cover letter. Guidelines for resubmitting your figure files are available below the reviewer comments at the end of this letter.

If applicable, we recommend that you deposit your laboratory protocols in protocols.io to enhance the reproducibility of your results. Protocols.io assigns your protocol its own identifier (DOI) so that it can be cited independently in the future. For instructions see: https://journals.plos.org/plosone/s/submission-guidelines#loc-laboratory-protocols. Additionally, PLOS ONE offers an option for publishing peer-reviewed Lab Protocol articles, which describe protocols hosted on protocols.io. Read more information on sharing protocols at https://plos.org/protocols?utm_medium=editorial-email&utm_source=authorletters&utm_campaign=protocols.

As the corresponding author, your ORCID iD is verified in the submission system and will appear in the published article. PLOS supports the use of ORCID, and we encourage all coauthors to register for an ORCID iD and use it as well. Please encourage your coauthors to verify their ORCID iD within the submission system before final acceptance, as unverified ORCID iDs will not appear in the published article. *Only*  the individual author can complete the verification step; PLOS staff *cannot*  verify ORCID iDs on behalf of authors.

We look forward to receiving your revised manuscript.

Kind regards,

Srinivasa Reddy Bonam

Academic Editor

PLOS One

Journal Requirements:

-->When submitting your revision, we need you to address these additional requirements.

1. Please ensure that your manuscript meets PLOS ONE's style requirements, including those for file naming. The PLOS ONE style templates can be found at

https://journals.plos.org/plosone/s/file?id=wjVg/PLOSOne_formatting_sample_main_body.pdf and

https://journals.plos.org/plosone/s/file?id=ba62/PLOSOne_formatting_sample_title_authors_affiliations.pdf

2. To comply with PLOS One submissions requirements, in your Methods section, please provide additional information regarding the experiments involving animals and ensure you have included details on (1) methods of sacrifice, (2) methods of anesthesia and/or analgesia, and (3) efforts to alleviate suffering.

3. Thank you for stating the following in the Competing Interests section:

“Yo Muraki, Yuumi Okuzono, Rina Kurisu, Akiko Abiru, and Chihiro Take are employees of Takeda Pharmaceutical Company Limited. Koji Tamada and Yukimi Sakoda hold stocks in Noile-Immune Biotech Inc. and receive remuneration from Noile-Immune Biotech Inc. In addition, Yukimi Sakoda received research funding from Noile-Immune Biotech Inc.”

We note that you received funding from a commercial source: Noile-Immune Biotech Inc

Please provide an amended Competing Interests Statement that explicitly states this commercial funder, along with any other relevant declarations relating to employment, consultancy, patents, products in development, marketed products, etc.

Within this Competing Interests Statement, please confirm that this does not alter your adherence to all PLOS ONE policies on sharing data and materials by including the following statement: ""This does not alter our adherence to PLOS ONE policies on sharing data and materials.” (as detailed online in our guide for authors http://journals.plos.org/plosone/s/competing-interests).  If there are restrictions on sharing of data and/or materials, please state these. Please note that we cannot proceed with consideration of your article until this information has been declared.

Please include your amended Competing Interests Statement within your cover letter. We will change the online submission form on your behalf.

4. When completing the data availability statement of the submission form, you indicated that you will make your data available on acceptance. We strongly recommend all authors decide on a data sharing plan before acceptance, as the process can be lengthy and hold up publication timelines. Please note that, though access restrictions are acceptable now, your entire data will need to be made freely accessible if your manuscript is accepted for publication. This policy applies to all data except where public deposition would breach compliance with the protocol approved by your research ethics board. If you are unable to adhere to our open data policy, please kindly revise your statement to explain your reasoning and we will seek the editor's input on an exemption. Please be assured that, once you have provided your new statement, the assessment of your exemption will not hold up the peer review process.

5. Thank you for stating the following in the Competing Interests section:

“Yo Muraki, Yuumi Okuzono, Rina Kurisu, Akiko Abiru, and Chihiro Take are employees of Takeda Pharmaceutical Company Limited. Koji Tamada and Yukimi Sakoda hold stocks in Noile-Immune Biotech Inc. and receive remuneration from Noile-Immune Biotech Inc. In addition, Yukimi Sakoda received research funding from Noile-Immune Biotech Inc.”

We note that one or more of the authors are employed by a commercial company:

• Takeda Pharmaceutical Company Limited

• Noile-Immune Biotech Inc.

1) Please provide an amended Funding Statement declaring this commercial affiliation, as well as a statement regarding the Role of Funders in your study. If the funding organization did not play a role in the study design, data collection and analysis, decision to publish, or preparation of the manuscript and only provided financial support in the form of authors' salaries and/or research materials, please review your statements relating to the author contributions, and ensure you have specifically and accurately indicated the role(s) that these authors had in your study. You can update author roles in the Author Contributions section of the online submission form.

Please also include the following statement within your amended Funding Statement.

“The funder provided support in the form of salaries for authors [insert relevant initials], but did not have any additional role in the study design, data collection and analysis, decision to publish, or preparation of the manuscript. The specific roles of these authors are articulated in the ‘author contributions’ section.”

If your commercial affiliation did play a role in your study, please state and explain this role within your updated Funding Statement.

2) Please also provide an updated Competing Interests Statement declaring this commercial affiliation along with any other relevant declarations relating to employment, consultancy, patents, products in development, or marketed products, etc.

Within your Competing Interests Statement, please confirm that this commercial affiliation does not alter your adherence to all PLOS ONE policies on sharing data and materials by including the following statement: ""This does not alter our adherence to  PLOS ONE policies on sharing data and materials.” (as detailed online in our guide for authors http://journals.plos.org/plosone/s/competing-interests) . If this adherence statement is not accurate and  there are restrictions on sharing of data and/or materials, please state these. Please note that we cannot proceed with consideration of your article until this information has been declared.

Please include both an updated Funding Statement and Competing Interests Statement in your cover letter. We will change the online submission form on your behalf.

6. If the reviewer comments include a recommendation to cite specific previously published works, please review and evaluate these publications to determine whether they are relevant and should be cited. There is no requirement to cite these works unless the editor has indicated otherwise.

7. Please review your reference list to ensure that it is complete and correct. If you have cited papers that have been retracted, please include the rationale for doing so in the manuscript text, or remove these references and replace them with relevant current references. Any changes to the reference list should be mentioned in the rebuttal letter that accompanies your revised manuscript. If you need to cite a retracted article, indicate the article’s retracted status in the References list and also include a citation and full reference for the retraction notice.

Reviewers' comments:

Reviewer's Responses to Questions

**Comments to the Author**

1. Is the manuscript technically sound, and do the data support the conclusions?

The manuscript must describe a technically sound piece of scientific research with data that supports the conclusions. Experiments must have been conducted rigorously, with appropriate controls, replication, and sample sizes. The conclusions must be drawn appropriately based on the data presented.

Reviewer #1: Partly

Reviewer #2: Yes

**********

2. Has the statistical analysis been performed appropriately and rigorously?

Reviewer #1: Yes

Reviewer #2: Yes

**********

3. Have the authors made all data underlying the findings in their manuscript fully available?

The PLOS Data policy requires authors to make all data underlying the findings described in their manuscript fully available without restriction, with rare exception (please refer to the Data Availability Statement in the manuscript PDF file). The data should be provided as part of the manuscript or its supporting information, or deposited to a public repository. For example, in addition to summary statistics, the data points behind means, medians and variance measures should be available. If there are restrictions on publicly sharing data—e.g. participant privacy or use of data from a third party—those must be specified.

Reviewer #1: Yes

Reviewer #2: Yes

**********

4. Is the manuscript presented in an intelligible fashion and written in standard English?

PLOS ONE does not copyedit accepted manuscripts, so the language in submitted articles must be clear, correct, and unambiguous. Any typographical or grammatical errors should be corrected at revision, so please note any specific errors here.

Reviewer #1: Yes

Reviewer #2: Yes

**********

5. Review Comments to the Author

Please use the space provided to explain your answers to the questions above. You may also include additional comments for the author, including concerns about dual publication, research ethics, or publication ethics. (Please upload your review as an attachment if it exceeds 20,000 characters)

Reviewer #1: The manuscript titled Gene Expression Profiling of Recipient Immune Cells Induced by 7 × 19 CAR-T Cell Dosing in a Syngeneic Mouse Model” by Yo Muraki et al. discusses the underlying mechanism of action of 7 x 19 CAR-T cells reported earlier. Earlier, the 7 x 19 CAR-T cells had shown the dependency on resident immune cells for their activity. Therefore, the paper focuses on the cellular composition and gene expression patterns of resident immune cells during the early tumour-induction phase in solid tumours, in the presence of 7 x 19 CAR-T cells to gain insights into the possible mechanism of action.

The experimental approach is good, but the data analysis and figure presentation may need further refinement. A more stringent criterion, like at least a 2-fold change, is suggested to be used for differentially expressed genes. The manuscript needs further refinement as suggested in the reviewer's report.

Reviewer #2: 1. In Figure 1, the authors show improved tumor reduction with 7×19 CAR-T cells compared to conventional CAR-T cells. However, no survival analysis is provided. Without survival data, it is difficult to assess the long-term therapeutic benefit of this treatment.

2. In Figure 2, the authors report increased percentages of CD8+ T cells and macrophages in the tumor microenvironment. However, only percentage data are presented, and absolute cell counts are not shown. This makes it unclear whether there is a true increase in immune cell numbers or only a relative change.

3. The study reports upregulation of multiple chemokines and immune-related pathways, but these findings are based only on transcriptomic analysis. No protein-level validation (such as ELISA) or functional assays are provided to confirm these observations.

4. The individual roles of IL-7 and CCL19 are not separately analyzed. Since both are expressed together in the CAR-T construct, it is unclear which factor is primarily responsible for the observed immunological effects.

**********

6. PLOS authors have the option to publish the peer review history of their article (what does this mean?). If published, this will include your full peer review and any attached files.

If you choose “no”, your identity will remain anonymous but your review may still be made public.

**Do you want your identity to be public for this peer review?**  For information about this choice, including consent withdrawal, please see our Privacy Policy.

Reviewer #1: **Yes:** Khalid Hussain Bhat, PhD. SKUAST-K, India.

Reviewer #2: **Yes:** Apoorva Rajput

**********

[NOTE: If reviewer comments were submitted as an attachment file, they will be attached to this email and accessible via the submission site. Please log into your account, locate the manuscript record, and check for the action link "View Attachments". If this link does not appear, there are no attachment files.]

To ensure your figures meet our technical requirements, please review our figure guidelines: https://journals.plos.org/plosone/s/figures

You may also use PLOS’s free figure tool, NAAS, to help you prepare publication quality figures: https://journals.plos.org/plosone/s/figures#loc-tools-for-figure-preparation.

NAAS will assess whether your figures meet our technical requirements by comparing each figure against our figure specifications.

Attachment Submitted filename: PONE-D-26-00663 Comments_Khalid.docx

Attachment Submitted filename: Review 1.docx

# Author comment: Author response to Decision Letter 1

**DOI:** 10.1371/journal.pone.0352813.r003

26 May 2026

5/22/2026

Emily Chenette

Editor-in-Chief

PLOS One

Dear Editor:

Thank you for your comment on our submission entitled “Gene Expression Profiling of Recipient Immune Cells Induced by 7 × 19 CAR-T Cell Dosing in a Syngeneic Mouse Model.” We have revised the manuscript accordingly and present our response to the comment below. The changes to the manuscript are highlighted in yellow in the Track Changes version of the manuscript file.

Response to editor

Comment:

1. Please ensure that your manuscript meets PLOS ONE's style requirements, including those for file naming. The PLOS ONE style templates can be found at

https://journals.plos.org/plosone/s/file?id=wjVg/PLOSOne_formatting_sample_main_body.pdf and

https://journals.plos.org/plosone/s/file?id=ba62/PLOSOne_formatting_sample_title_authors_affiliations.pdf

Response:

We confirmed that the manuscript meets PLOS ONE’s tyle requirements.

Comment:

2. To comply with PLOS One submissions requirements, in your Methods section, please provide additional information regarding the experiments involving animals and ensure you have included details on (1) methods of sacrifice, (2) methods of anesthesia and/or analgesia, and (3) efforts to alleviate suffering.

Response:

Thank you for this comment. To comply with the PLOS ONE requirements for animal experiments, we have revised the Methods section to include additional details on (1) the method of sacrifice/euthanasia, (2) anesthesia and/or analgesia, and (3) efforts to alleviate suffering. The mice were anesthetized with isoflurane (1%–5%) and euthanized under deep isoflurane anesthesia. We also added a description of our efforts to reduce pain and distress, including the use of predefined humane endpoint criteria approved by the Institutional Animal Care and Use Committee (IACUC); animals were euthanized immediately upon reaching these endpoints.

Comment:

3. Thank you for stating the following in the Competing Interests section:

“Yo Muraki, Yuumi Okuzono, Rina Kurisu, Akiko Abiru, and Chihiro Take are employees of Takeda Pharmaceutical Company Limited. Koji Tamada and Yukimi Sakoda hold stocks in Noile-Immune Biotech Inc. and receive remuneration from Noile-Immune Biotech Inc. In addition, Yukimi Sakoda received research funding from Noile-Immune Biotech Inc.”

We note that you received funding from a commercial source: Noile-Immune Biotech Inc

Please provide an amended Competing Interests Statement that explicitly states this commercial funder, along with any other relevant declarations relating to employment, consultancy, patents, products in development, marketed products, etc.

Response:

Thank you for this comment. We have amended the Competing Interests statement to explicitly declare the commercial funder, Noile-Immune Biotech Inc., and added this information to the Funding Statement. The revised Competing Interests statement now reads as follows:

YM, YO, RK, AA, and CT are employees of Takeda Pharmaceutical Company Limited. KT and YS hold stocks in Noile-Immune Biotech Inc. and receive remuneration from Noile-Immune Biotech Inc. In addition, Yukimi Sakoda received research funding from Noile-Immune Biotech Inc. This does not alter our adherence to PLOS ONE policies on sharing data and materials (as detailed online in our guide for authors http://journals.plos.org/plosone/s/competing-interests).

Comment:

Within this Competing Interests Statement, please confirm that this does not alter your adherence to all PLOS ONE policies on sharing data and materials by including the following statement: ""This does not alter our adherence to PLOS ONE policies on sharing data and materials.” (as detailed online in our guide for authors http://journals.plos.org/plosone/s/competing-interests). If there are restrictions on sharing of data and/or materials, please state these. Please note that we cannot proceed with consideration of your article until this information has been declared.

Please include your amended Competing Interests Statement within your cover letter. We will change the online submission form on your behalf.

Response:

Thank you for this request. We have added the following statement to the amended Competing Interests Statement in the revised cover letter: “This does not alter our adherence to PLOS ONE policies on sharing data and materials.”

Comment:

4. When completing the data availability statement of the submission form, you indicated that you will make your data available on acceptance. We strongly recommend all authors decide on a data sharing plan before acceptance, as the process can be lengthy and hold up publication timelines. Please note that, though access restrictions are acceptable now, your entire data will need to be made freely accessible if your manuscript is accepted for publication. This policy applies to all data except where public deposition would breach compliance with the protocol approved by your research ethics board. If you are unable to adhere to our open data policy, please kindly revise your statement to explain your reasoning and we will seek the editor's input on an exemption. Please be assured that, once you have provided your new statement, the assessment of your exemption will not hold up the peer review process.

Response:

Thank you for this comment. We have deposited the data in the NCBI Gene Expression Omnibus (GEO) under accession numbers GSE313034 and GSE314885. The datasets will be made publicly available upon acceptance of the manuscript for publication.

Comment:

5. Thank you for stating the following in the Competing Interests section:

“Yo Muraki, Yuumi Okuzono, Rina Kurisu, Akiko Abiru, and Chihiro Take are employees of Takeda Pharmaceutical Company Limited. Koji Tamada and Yukimi Sakoda hold stocks in Noile-Immune Biotech Inc. and receive remuneration from Noile-Immune Biotech Inc. In addition, Yukimi Sakoda received research funding from Noile-Immune Biotech Inc.”

We note that one or more of the authors are employed by a commercial company:

• Takeda Pharmaceutical Company Limited

• Noile-Immune Biotech Inc.

1) Please provide an amended Funding Statement declaring this commercial affiliation, as well as a statement regarding the Role of Funders in your study. If the funding organization did not play a role in the study design, data collection and analysis, decision to publish, or preparation of the manuscript and only provided financial support in the form of authors' salaries and/or research materials, please review your statements relating to the author contributions, and ensure you have specifically and accurately indicated the role(s) that these authors had in your study. You can update author roles in the Author Contributions section of the online submission form.

Please also include the following statement within your amended Funding Statement.

“The funder provided support in the form of salaries for authors [insert relevant initials], but did not have any additional role in the study design, data collection and analysis, decision to publish, or preparation of the manuscript. The specific roles of these authors are articulated in the ‘author contributions’ section.”

If your commercial affiliation did play a role in your study, please state and explain this role within your updated Funding Statement.

Response:

Thank you for this comment. We have amended the Funding Statement to declare the commercial affiliations and include a statement regarding the role of the funders. We added the following text to the revised cover letter:

The Takeda Pharmaceutical Company Limited provided support in the form of salaries for YM, YO, RK, AA, CT, Noile-Immune Biotech Inc provided support in the form of remuneration for KT, YS, but did not play any additional role in the study design, data collection and analysis, decision to publish, or preparation of the manuscript. The specific roles of these authors are articulated in the ‘Author Contributions’ section.

In addition, we have updated the author roles in the Author Contributions section of the online submission form to ensure they are specific and accurate.

Comment:

2) Please also provide an updated Competing Interests Statement declaring this commercial affiliation along with any other relevant declarations relating to employment, consultancy, patents, products in development, or marketed products, etc.

Within your Competing Interests Statement, please confirm that this commercial affiliation does not alter your adherence to all PLOS ONE policies on sharing data and materials by including the following statement: ""This does not alter our adherence to PLOS ONE policies on sharing data and materials.” (as detailed online in our guide for authors http://journals.plos.org/plosone/s/competing-interests). If this adherence statement is not accurate and there are restrictions on sharing of data and/or materials, please state these. Please note that we cannot proceed with consideration of your article until this information has been declared.

Please include both an updated Funding Statement and Competing Interests Statement in your cover letter. We will change the online submission form on your behalf.

Response:

Thank you for this request. We have included an updated Funding Statement and an updated Competing Interests Statement in our revised cover letter. The Competing Interests Statement also includes the following sentence: “This does not alter our adherence to PLOS ONE policies on sharing data and materials.”

Comment:

6. If the reviewer comments include a recommendation to cite specific previously published works, please review and evaluate these publications to determine whether they are relevant and should be cited. There is no requirement to cite these works unless the editor has indicated otherwise.

Response:

Thank you for this comment. The reviewer comments did not include any recommendation to cite specific previously published works. Therefore, no additional citations were added in response to this point.

Comment:

7. Please review your reference list to ensure that it is complete and correct. If you have cited papers that have been retracted, please include the rationale for doing so in the manuscript text, or remove these references and replace them with relevant current references. Any changes to the reference list should be mentioned in the rebuttal letter that accompanies your revised manuscript. If you need to cite a retracted article, indicate the article’s retracted status in the References list and also include a citation and full reference for the retraction notice.

Response:

Thank you for this comment. We carefully reviewed the reference list and confirmed that none of the cited papers have been retracted. Therefore, no changes were made to the reference list in response to this point.

Response to reviewer

Reviewer #1: The manuscript titled Gene Expression Profiling of Recipient Immune Cells Induced by 7 × 19 CAR-T Cell Dosing in a Syngeneic Mouse Model” by Yo Muraki et al. discusses the underlying mechanism of action of 7 x 19 CAR-T cells reported earlier. Earlier, the 7 x 19 CAR-T cells had shown the dependency on resident immune cells for their activity. Therefore, the paper focuses on the cellular composition and gene expression patterns of resident immune cells during the early tumor-induction phase in solid tumors, in the presence of 7 x 19 CAR-T cells to gain insights into the possible mechanism of action.

The experimental approach is good, but the data analysis and figure presentation may need further refinement. A more stringent criterion, like at least a 2-fold change, is suggested to be used for differentially expressed genes. The manuscript needs further refinement as suggested in the reviewer's report.

Following minor changes need to be included,

Comment:

Page 3 Line 34: change transcriptomics to transcriptomic

Response:

We changed “transcriptomics” to “transcriptomic.” (Page2 Line 35)

Comment:

Page 12 Line 313: I wonder if 1.5-fold change in expression level will have a significant biological impact. A more stringent criteria like at least 2-fold change is suggested to be used.

Response:

For the AmpliSeq analysis, we changed the threshold for differentially expressed genes from 1.5- to 2-fold. Accordingly, we updated the volcano plot and repeated the pathway enrichment analysis (Figs. 3 and 4). In the single-cell RNA seq study, we attempted to understand the whole cellular transcriptomic change following 7 × 19 CAR-T dosing and considered that the information including gene with low change is important for pathway enrichment analysis. Thus, we set 1.5-fold change as criteria in the single-cell RNA seq to capture broader transcriptomic changes. We described this point in the manuscript (Page 18, Line 401-405).

Comment:

Page 19 Line 418-420: The transcriptomic landscape cannot be called markedly different with a threshold of 1.5 fold being set. Calling it a modest change would be more appropriate.

Response:

We removed the word “markedly.” (Page 21. Line 474)

Comment:

Figures:

In all figures a succinct experimental procedure should be mentioned for ease and clarity.

Response:

We added a succinct description of the experimental procedure to all figure panels/legends for clarity.

Comment:

Figure 1 B and C: Label the diagram with Marker rather than the fluorophore. Gating strategy should be succinctly mentioned in the figure legend

Response:

We relabeled the diagram using marker names (instead of fluorophores) in Figure 1B and 1D and added a succinct description of the gating strategy to the figure legend.

Comment:

Figure 1 D: the schematic is not a true representation of study design as the tumor volume data points at 0 and 5 days are represented in Figure 1 E and F but the same is not indicated in the schematic Figure 1 D.

Response:

We revised the schematic and consolidated Figure 1E and 1F to accurately reflect the study design, including the tumor volume measurement time points.

Comment:

Figure 2: Gating strategy should be succinctly mentioned in the figure legend

E: please indicate the exact p value when comparing “no treatment” vs “7 x 19 CART-T”

F: please indicate whether statistics between “Vehicle” vs “7 x 19 CART-T” was significant.

Response:

We added a succinct description of the gating strategy to the Figure 2 legend.

In Figure 2E, we added the exact p-value for the comparison between “No treatment” and “7 × 19 CAR-T.”

In Figure 2F, we elucidated that the difference between “Vehicle” and “7 × 19 CAR-T” was not statistically significant and updated the figure accordingly.

Comment:

Figure 3: It was difficult to make out anything out of the data presented given the low quality pictures provided.

Figure 4: It was difficult to make out anything out of the data presented given the low quality pictures provided.

Response:

We revised Figures 3 and 4 to improve figure quality and readability.

Comment:

Figure 5: abbreviation CPM on Y-axis should be expanded in the legend.

Response:

We expanded “CPM” in the legend as “counts per minute.”

Comment:

Suggested Experiments:

Overall, the paper gives an indication that treatment with 7 x 19 CAR-T cells induces a proinflammatory response in the tumors at the early stages with increased infiltration of immune cells. This fact can be cross-checked by using histopathological examinations of the tumor at early stages. Furthermore, validating the pro-inflammatory response through circulating cytokines in the mouse circulatory system would add a further degree of validation and confidence.

Response:

Thank you for this comment regarding the study design. As you suggested, histopathological examination of tumors at early time points and measurement of circulating cytokines would provide additional validation and confidence in the proposed pro-inflammatory response. The present study focused on elucidating transcriptomic changes induced by the 7 × 19 CAR-T treatment; therefore, we did not perform these additional assays. We have added this limitation and the need for these follow-up analyses to the Discussion section (Page 23, Line 520-528).

Reviewer #2:

Comment:

1. In Figure 1, the authors show improved tumor reduction with 7 × 19 CAR-T cells compared

Attachment Submitted filename: Response to Reviewers_260522-2.docx

# Editor report: Decision Letter 1

**DOI:** 10.1371/journal.pone.0352813.r004

**Authors:** Srinivasa Reddy Bonam, Srinivasa Reddy Bonam

15 Jun 2026

Gene Expression Profiling of Recipient Immune Cells Induced by 7 × 19 CAR-T Cell Dosing in a Syngeneic Mouse Model

PONE-D-26-00663R1

Dear Dr. Chihiro Take,

We’re pleased to inform you that your manuscript has been judged scientifically suitable for publication and will be formally accepted for publication once it meets all outstanding technical requirements.

Within one week, you’ll receive an e-mail detailing the required amendments. When these have been addressed, you’ll receive a formal acceptance letter and your manuscript will be scheduled for publication.

An invoice will be generated when your article is formally accepted. Please note, if your institution has a publishing partnership with PLOS and your article meets the relevant criteria, all or part of your publication costs will be covered. Please make sure your user information is up-to-date by logging into Editorial Manager at Editorial Manager® and clicking the ‘Update My Information' link at the top of the page. For questions related to billing, please contact billing support.

If your institution or institutions have a press office, please notify them about your upcoming paper to help maximize its impact. If they’ll be preparing press materials, please inform our press team as soon as possible -- no later than 48 hours after receiving the formal acceptance. Your manuscript will remain under strict press embargo until 2 pm Eastern Time on the date of publication. For more information, please contact onepress@plos.org.

Kind regards,

Srinivasa Reddy Bonam

Academic Editor

PLOS One

Additional Editor Comments (optional):

Reviewers' comments:

# Editor report: Acceptance letter

**DOI:** 10.1371/journal.pone.0352813.r005

**Authors:** Srinivasa Reddy Bonam, Srinivasa Reddy Bonam

PONE-D-26-00663R1

PLOS One

Dear Dr. Take,

I'm pleased to inform you that your manuscript has been deemed suitable for publication in PLOS One. Congratulations! Your manuscript is now being handed over to our production team.

At this stage, our production department will prepare your paper for publication. This includes ensuring the following:

* All references, tables, and figures are properly cited

* All relevant supporting information is included in the manuscript submission,

* There are no issues that prevent the paper from being properly typeset

You will receive further instructions from the production team, including instructions on how to review your proof when it is ready. Please keep in mind that we are working through a large volume of accepted articles, so please give us a few days to review your paper and let you know the next and final steps.

Lastly, if your institution or institutions have a press office, please let them know about your upcoming paper now to help maximize its impact. If they'll be preparing press materials, please inform our press team within the next 48 hours. Your manuscript will remain under strict press embargo until 2 pm Eastern Time on the date of publication. For more information, please contact onepress@plos.org.

You will receive an invoice from PLOS for your publication fee after your manuscript has reached the completed accept phase. If you receive an email requesting payment before acceptance or for any other service, this may be a phishing scheme. Learn how to identify phishing emails and protect your accounts at https://explore.plos.org/phishing.

If we can help with anything else, please email us at customercare@plos.org.

Thank you for submitting your work to PLOS ONE and supporting open access.

Kind regards,

PLOS ONE Editorial Office Staff

on behalf of

Dr. Srinivasa Reddy Bonam

Academic Editor

PLOS One
